# Br-Induced Suppression of Low-Temperature Phase Transitions in Mixed-Cation Mixed-Halide Perovskites

**DOI:** 10.1021/acs.chemmater.4c01670

**Published:** 2024-10-04

**Authors:** Juanita Hidalgo, Joachim Breternitz, Daniel M. Többens, Diana K. LaFollette, Charles N. B. Pedorella, Meng-Ju Sher, Susan Schorr, Juan-Pablo Correa-Baena

**Affiliations:** † School of Materials Science and Engineering, Georgia Institute of Technology, Atlanta, Georgia 30332, United States; ‡ Department Structure and Dynamics of Energy Materials, Helmholtz-Zentrum Berlin Für Materialien und Energie, Hahn-Meitner-Platz 1, Berlin 14109, Germany; § Department of Physics, Wesleyan University, Middletown, Connecticut 06459, United States; ∥ Freie Universitaet Berlin, Institute of Geological Sciences, Malteser Str. 74-200, Berlin 12249, Germany

## Abstract

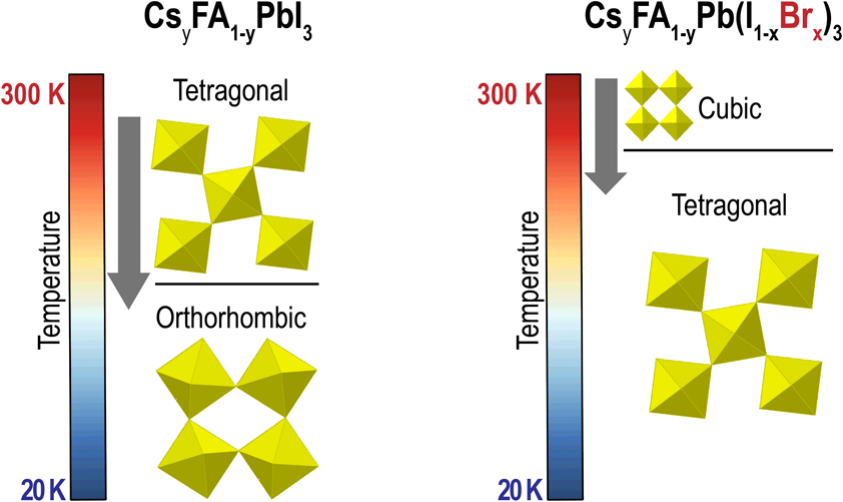

Mixed-cation mixed-halide lead perovskites have been shown to be excellent candidates for solar energy conversion. However, understanding the structural phases of these mixed-ion perovskites across a wide range of operating temperatures, including very low temperatures for space applications, is crucial. In this study, we investigated the structure of formamidinium-based Cs_*y*_FA_1–*y*_Pb(Br_*x*_I_1–*x*_)_3_ using low-temperature in situ synchrotron powder X-ray diffraction. Our findings revealed that substituting the I anion with Br in mixed-cation (Cs,FA) perovskites suppressed the phase transformation from tetragonal to orthorhombic at low temperatures. The addition of Br also prevented the formation of nonperovskite secondary phases. We gained fundamental insights into the structural behavior of these materials by creating a low-temperature phase diagram for the compositional set of mixed-cation mixed-halides. This understanding of the structural properties lays the groundwork for designing more robust and efficient energy materials capable of functioning under extreme temperature conditions, including space-based solar energy conversion.

## Introduction

Mixed-cation mixed-halide lead halide perovskites (LHP) have emerged as promising materials for solar energy conversion.^1^ Specifically, formamidinium (FA)-rich compositions have demonstrated higher thermal stability and an optimal bandgap for single-junction solar cells.^2^ Compositional mixing has been employed to achieve optimal optoelectronic properties and stability.^3,4^ However, it is crucial to unravel the structural phases across a wide range of operating temperatures, including very low temperatures relevant to space applications, to ensure the proper functioning and stability of these materials. The most common and efficient compositions include mixing cesium (Cs) and FA in the A-site as well as iodine (I) and bromine (Br) in the X-site, resulting in (Cs,FA)Pb(I,Br)_3_ LHP compositions. A knowledge gap exists on a complete phase diagram for both mixed-cation and mixed-halide compositions. In this work, we aim to construct a low-temperature phase diagram for (Cs,FA) mixed halide (I,Br) perovskites.

Previous studies have reported low-temperature phase diagrams for single-ion compositions such as FAPbI_3_,^5^ CsPbI_3_,^6^ FAPbBr_3_,^7^ and CsPbBr_3_.^8^ Generally, perovskites exhibit a cubic (α) phase at high temperatures above a certain threshold. As the temperature decreases, the perovskite structure undergoes phase transitions from cubic to tetragonal (β) and then from tetragonal to orthorhombic (γ) phase. Phase diagrams have also been studied for single-cation/mixed-halide or mixed-cation/single-halide compositions. For instance, Lehmann et al. investigated and presented the temperature phase diagram for MAPb(I,Br)_3_ compositions, highlighting the two phase transitions.^9^ Nasstrom et al. reported the high-temperature phase diagram for CsPb(I,Br)_3_, observing different phase transitions during heating and cooling.^10^ Other studies focused on the phase diagram for the mixed-cation iodide-based perovskites, either (MA,FA)PbI_3_^11^ or (Cs,FA)PbI_3_.^12,13^ In the first,^11^ they used Raman spectroscopy and photoluminescence to uncover different low-temperature phase transitions dependent on the FA content, related to its higher symmetry compared to MA. In the latter, for (Cs,FA) perovskite, the authors revealed the cubic-tetragonal-orthorhombic transitions and solubility limit of the Cs/FA ratio to form a pure perovskite phase.^12,13^

In the case of pure Br-based compositions, it has been demonstrated that Cs substitution in FAPbBr_3_ perovskite suppresses the orthorhombic phase transition at low temperatures, attributed to a geometric blocking associated with the rotation of FA molecules.^14^ The suppression of the orthorhombic phase at low temperatures has also been observed in mixed-cation MA-dimethylammonium (DMA) lead bromide perovskites (MA,DMA)PbBr_3_.^15,16^ This suppression was attributed to the increased disorder of the organic cations in the mixed samples, leading to higher symmetry and less distortion of the inorganic framework. The improved structural stability also enhanced their photodetection capabilities.^16^ Despite the knowledge from studying low-temperature phase transitions in various cation and halide combinations, a complete structural phase diagram for widely used and highly efficient LHPs (Cs,FA)Pb(I,Br)_3_ is still lacking.

Herein, we study the crystal structure of FA-based LHP compositions Cs_*y*_FA_1–*y*_Pb(Br_*x*_I_1–*x*_)_3_ by low-temperature in situ synchrotron powder X-ray diffraction (XRD) from 300 to 23 K. Our focus was on FA-rich and I-rich compositions to maintain a low bandgap suitable for single-junction solar cells. Our findings reveal that the low-temperature orthorhombic phase transition is suppressed as Br replaces I in the mixed-cation LHP compositions. Additionally, the inclusion of Br eliminates secondary nonperovskite phases. We construct three halide-dependent temperature phase diagrams for different Cs concentrations, demonstrating that adding Br to the mixed-cation (Cs,FA) system stabilizes the higher-symmetry perovskite structure from room to low temperatures and prevents the formation of nonperovskite phases. This fundamental understanding of the perovskite structure in a wide range of temperatures provides a basis for designing and developing more resilient and efficient materials capable of functioning under extreme conditions. Comprehending the state-of-the-art halide perovskite’s structure across the entire temperature range is paramount.

## Results and Discussion

### Properties and Structure at Room Temperature and 300 K

**Figure 1:**
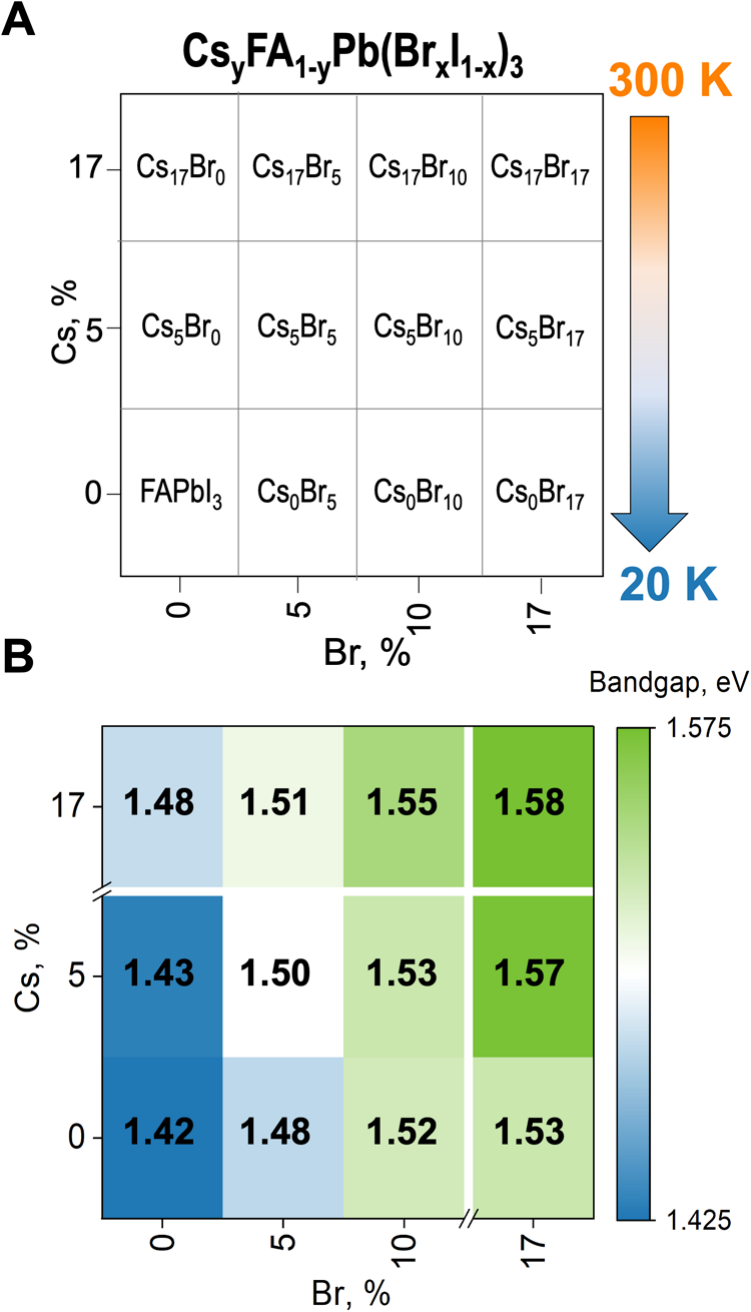
Mixed-cation and mixed-halide perovskite compositions of study. (A) Selected compositions to study the in situ phase transitions from 300 to 23 K and (B) bandgap from UV–vis at room temperature.

To investigate the low-temperature phase transitions for different FA-based LHP compositions, we selected a mixed-cation and mixed-halide compositional space Cs_*y*_FA_1–*y*_Pb(Br_*x*_I_1–*x*_)_3_, as shown in Figure 1A. We chose this compositional space to have FA-rich compositions with ideal bandgaps for single-junction solar cells.^2,5,17^ The selected upper limit for Cs was 17% molar, given that its thermodynamic limit to form a single-phase perovskite when mixed with FA is around 15^12,13,18^ and 20% molar.^19^ Therefore, we chose 17%, targeting a single-phase perovskite formation. The upper limit chosen for Br was 17%, keeping an I-based composition and equaling the maximum added percent of Cs.

Bandgap tunability is one of the main advantages of LHPs.^20^ To analyze the optoelectronic properties of the synthesized materials, we measured UV–vis absorption spectroscopy (at room temperature) and used the Tauc-plot method (Figure S1) to calculate the optical bandgap.^21^ In Figure 1B, the lowest experimental bandgap is 1.42 eV for the pure FAPbI_3_, which is close but lower than other reported values from calculations (1.51 eV),^22^ absorbing in the range to achieve the maximum theoretical power conversion efficiency for single-junction solar cells according to the Shockley–Queisser limit.^23^ However, FAPbI_3_ perovskite is unstable at room temperature and ambient atmosphere, transforming into a yellow powder with a hexagonal structure, identified here as 2H. For this reason, pure FAPbI_3_ is not further analyzed in this work. The LHP’s bandgap increased as Cs and Br were added to the FA-based compositions (Figure 1B). Both cation and halide substitution in FAPbI_3_ reduces the crystal structure’s lattice parameters, resulting in increased bandgap, as described and experimentally shown for the complete set of (Cs,FA) and (I,Br) compositions by An and others.^20^ We observe that adding Br increases the bandgap further compared to the Cs addition. It has been demonstrated that the bandgap and lattice parameters of semiconducting materials correlate with the effective anion radius of the halides, in this case, Br.^24^ Whereas the A-site cation does not participate in the band edge states, it does influence the room temperature optical properties indirectly through changes to the structure by the octahedral tilting and arrangement.^16,25^ We highlight that one of the main advantages of LHPs is their property tunability through compositional engineering, as we see here on bandgap tunability when we add Cs and Br. In this case, the studied compositions have a bandgap from 1.43 to 1.58 eV, remaining in an ideal range for high-efficiency single-junction perovskite solar cells.

**Figure 2:**
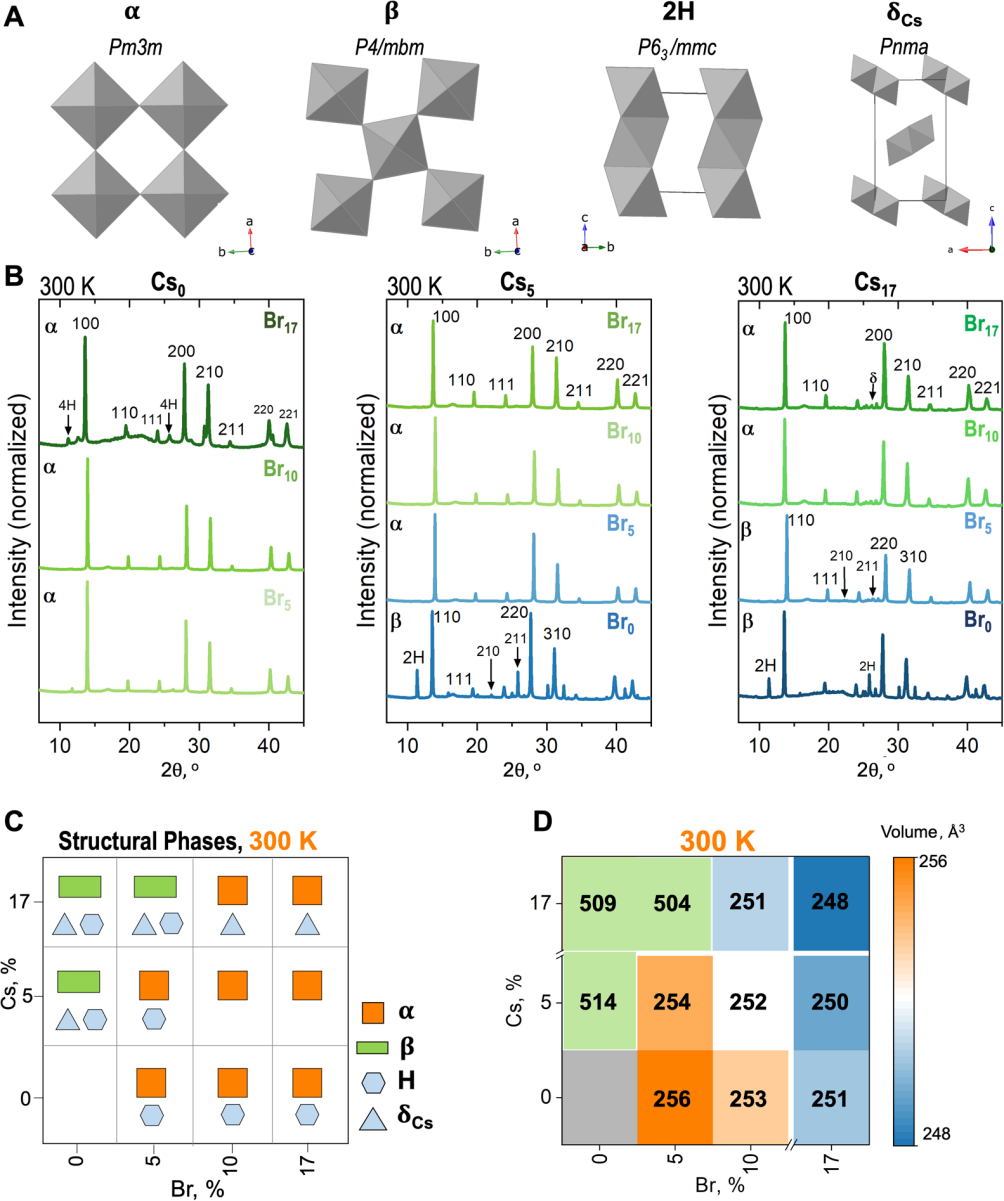
Crystal structure from powder XRD at 300 K. (A) Structural phases present in the (Cs,FA)Pb(I,Br)_3_ set of compositions. (B) XRD patterns for different Cs–Br concentrations for the (Cs,FA)–(I,Br) compositions at 300 K and vacuum, (C) structural phases from Le Bail refinements, and (D) unit cell volume for the (Cs,FA)–(I,Br) compositions at 300 K. (Cs,FA)Pb(I,Br)_3_ Temperature phase diagram.

To study the crystal structure of these LHPs, we performed powder XRD, as shown in Figure 2. In Figure 2A, we show the structure of the perovskite phase at 300 K, corresponding to either a cubic phase of space group *Pm*3̅*m* (α) or a tetragonal phase of space group *P*4/*mbm* (β). We analyzed in detail the XRD patterns at 300 K (Figure 2B) by Le Bail refinements (Figures S2–S4). From this initial analysis, we identified the structural phases present for each composition, as shown in Figure 2C, where α is an orange square and β is a green rectangle. All the studied compositions without Br, Cs_*x*_Br_0_, are β phase at 300 K. This structure is similar to the work by An and others,^20^ where (Cs_17_,FA_83_)PbI_3_ was tetragonal at room temperature, and with the work by Charles and co-workers,^12^ where (Cs_10_, FA_90_)PbI_3_ was tetragonal at 290 K. Adding Cs to FAPbI_3_ structure induces a chemical pressure or lattice strain effect due to the Cs and FA cation size mismatch, leading to a structural distortion, stronger FA-iodide hydrogen bonding interaction, and symmetry breaking.^26^ Therefore, the symmetry break when adding Cs explains the initial β phase for Cs_*x*_Br_0_. Adding only 5% Br to the Cs_17_ perovskite phase (Cs_17_Br_5_) also results in a β structure. This low amount of Br does not change the tetragonal structure, suggesting that since there is no octahedral tilting, there are also no changes in cation rotation. However, adding more Br (10 and 17%) leads to an initially cubic perovskite structure (α) at room temperature.^5^ Previous work by Johnston and co-workers showed that adding up to 15% Br to (Cs,MA,FA)PbI_3_ suppressed the organic cation rotation, enhancing long-lived carrier lifetimes. In this case, we suggest that adding 10 or 17% of Br would similarly cause a decrease in cation rotation, leading to a higher symmetry α perovskite.

Beyond the perovskite structural analysis, we also identified secondary nonperovskite phases from the powder XRDs in Figure 2. Single-cation CsPbI_3_ or FAPbI_3_ form nonperovskite phases with either face- or edge-sharing octahedra. Hexagonal face-sharing FAPbI_3_, referred to here as 2H, is a face-sharing octahedra structure with space group *P*6_3_/*mmc* and the orthorhombic edge-sharing CsPbI_3_, known as δ_Cs_, has a space group *Pnma* (Figure 2A).^27,28^ There are also other intermediate hexagonal polytypes between the corner-sharing and the pure face-sharing, known as 4H or 6H.^27,29^ These hexagonal polytypes (H) are present in mixed halides. Figure 2C summarizes the secondary phases at 300 K, showing the hexagonal polytypes H as hexagons and δ_Cs_ as triangles. All compositions Cs_0_Br_*x*_ show some hexagonal polytypes, specifically due to their characteristic diffraction peaks between 8 and 12° in 2θ of the diffraction pattern (Figure 2B). For Cs_5_Br_0_, we observe the formation of some nonperovskite δ_Cs_, possibly due to phase segregation.^12^ For Cs_5_Br_5_, some polytype H phases are observed, but remarkably, in Cs_5_Br_10_ and Cs_5_Br_17_, no secondary phases are formed. The work by Gratia and others^27^ shows that around 3% Cs is fundamental in FA-rich (I,Br) compositions to increase the structural chemical pressure, inhibiting the formation of polytypes, explaining why Cs is needed in (I,Br) compositions for improved stability and symmetry. Additionally, the work by Li,^13^ Charles,^12^ Mozur,^14^ and Ghosh,^26^ shows the importance of adding Cs up to 15–20% (solubility limit) since above this value, the mixed-cation LHP will phase segregate into their to phase single-cation nonperovskite phases. In the case of Cs_17_Br_*x*_, we overcome this solubility limit. Therefore, all Cs_17_Br_*x*_ compositions show the presence of the δ_Cs_ phase (Figure 2B). Herein, we see how a combination of adding less than 17% Cs and more than 5% Br (Cs_5_Br_10_ and Cs_5_Br_17_), in both cases, leads to the desired combination for a single-phase high symmetry structure.

Lastly, from the powder XRD at 300 K and the Le Bail analysis, we determined the lattice parameters and calculated the unit cell volume (Figure 2D). Tetragonal structures have lattice parameters *a* = *b* ≠ *c*. In the case of cubic structures, the lattice parameters are all equal: *a* = *b* = *c*. The volume for the tetragonal structure is significantly larger given that the values of *a* and *b* are around 8.96 Å, increasing the overall volume of the tetragonal unit cell. For the cubic unit cell volumes, we observe that the volume decreases as Cs and Br are added in FAPbI_3_. Given the replacement by both smaller-size ions, the overall cubic unit cell volume is expected to decrease. A smaller sized cation, Cs^+^, is replacing the larger cation FA^+^ as the halide Br^–^ is replacing a larger ion, I^–^.

**Figure 3:**
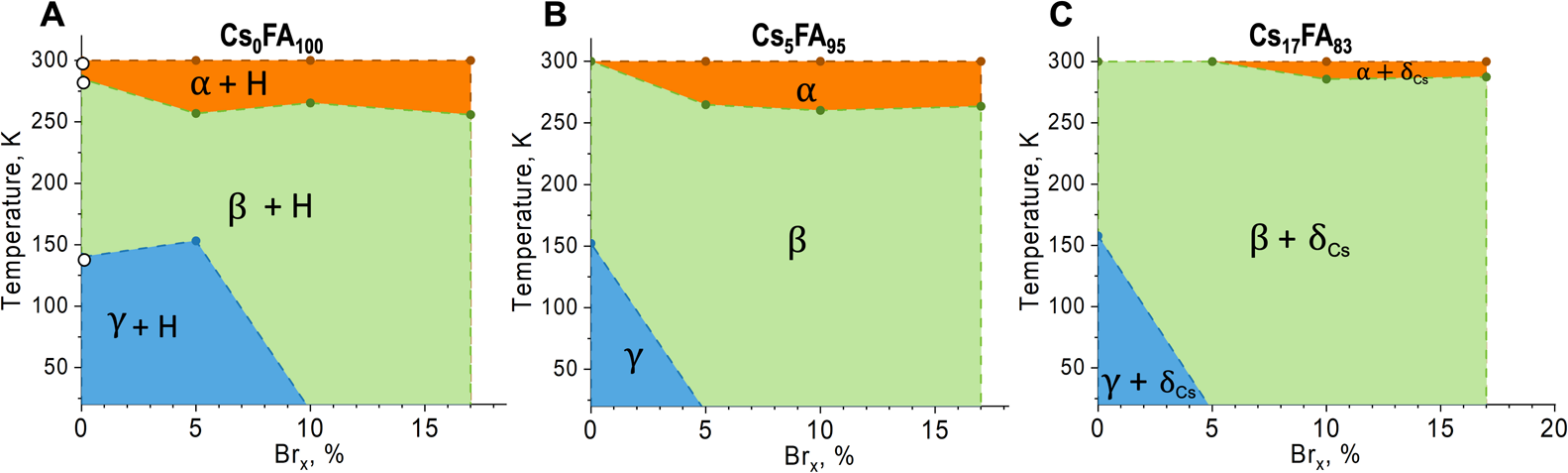
Composition–temperature phase diagram. Mixed-halide mixed-cation LHP in the (Cs,FA)Pb(I,Br)_3_ compositional space. (A) Without Cs (Cs_0_Br_*x*_) as a function of Br, (B) Cs 5% (Cs_5_Br_*x*_) as a function of Br, and (C) Cs 17% (Cs_17_Br_*x*_) as a function of Br. The lines are to guide the eye and highlight different phases.

To compose a low-temperature phase diagram for the (Cs,FA)–(I,Br) compositional space, we evaluated the in situ XRD of all samples by cooling the powders from 300 to 23 K in steps of 20 K (Figures S5–S7). From a general phase analysis, by plotting the diffraction pattern as a function of temperature (Figure S8), we identified the phase transition temperatures as new diffraction peaks appeared. These phase transition temperatures for all compositions are listed in Table S2. To show the overall mixed-cation and mixed-halide temperature phase diagram, we divided it into three for three Cs molar percentages, Cs_0_, Cs_5_, and Cs_17_ (Figure 3). Therefore, each temperature phase diagram is shown as a function of molar Br %.

First, Figure 3A shows the phase diagram for Cs_0_Br_*x*_. For Cs_0_Br_0_ and Cs_0_Br_5_, there are two low-temperature phase transitions, from α to β slightly above 250 K and from β to γ around 150 K. These two phase transitions have been reported for pure-iodide single-cation perovskites,^5−8^ mixed-cation (Cs,FA),^12^ and mixed-cation (MA,FA).^11,30^ Generally, it is expected for the perovskite to decrease its symmetry as it is cooled, as evidenced by the tilting of the corner-sharing octahedra from cubic to tetragonal and from tetragonal to orthorhombic. These transitions are also foreseen given that as the temperature decreases, the organic cations move slower and influence the octahedra tilting due to less movement. Moreover, when increasing the Br content to Cs_0_Br_10_ and Cs_0_Br_17_, we observe only one phase transition from α to β from 300 to 20 K. This represents higher stability of the tetragonal phase without the transition to orthorhombic, meaning there is less tilting of the octahedra. According to Johnston and co-workers,^31^ Br incorporation, up to around 15%, suppresses cation rotation in mixed-halide perovskites at room temperature. Therefore, we can explain that the suppressed cation rotation at room temperature keeps constant down to low temperatures. With respect to secondary phases, without Cs in FAPb(I,Br)_3_ (Cs_0_Br_*x*_), we observe the presence of hexagonal polytypes (H) in Figure S9 (explained in the previous section at 300 K).

Figure 3B shows the temperature phase diagram for Cs_0.05_FA_0.95_Pb(Br_*x*_I_1–*x*_)_3_ (Cs_5_Br_*x*_). For Cs_5_Br_0_, we observe the β phase at 300 K. We observe one phase transition from β to γ at 150 K. Adding Br, Cs_5_Br_5_ and Cs_5_Br_17_, leads to an initial α phase, indicating a higher perovskite symmetry at 300 K and discussed in the previous section of Figure 2. Cooling down the Cs_5_Br_*x*_ perovskites leads to one phase transitions from α to β slightly above 250 K, similar to the Cs_0_Br_*x*_ set. However, for all Cs_5_Br_*x*_ compositions, adding Br hinders the transition from β to γ, indicating better stability of the tetragonal phase and a higher perovskite symmetry at low temperatures such as 23 K. In general, for Cs_5_Br_*x*_, no secondary phases are observed (Figures S9–S10).

Figure 3C shows the temperature phase diagram for the case of Cs_0.17_FA_0.83_Pb(Br_*x*_I_1–*x*_)_3_ (Cs_17_Br_*x*_). Analogous to Cs_5_Br_0_, without Br Cs_17_Br_0_, the mixed-cation perovskite adopts the tetragonal structure at 300 K. This composition shows a phase transition from β to γ at around 150 K. At 300 K, adding Br, Cs_17_Br_5_, retains the tetragonal perovskite structure. Remarkably, this composition shows no phase transitions in the studied range from 300 to 23 K; the β perovskite phase is stable down to very low temperatures. Increasing the Br content (Cs_17_Br_10_ and Cs_17_Br_17_) leads to an initial α perovskite phase that will then transform to β phase at around 270 K.

Equivalent to the Cs_*y*_FA_1–*y*_Pb(Br_*x*_I_1–*x*_)_3_ phase with Cs_5_Br_*x*_, for Cs_17_Br_*x*_, we observe that adding Br suppresses the phase transition from β to γ all the way down to 23 K. Therefore, adding Br improves the stability and increases the symmetry of the perovskite structure at very low temperatures. Regarding the secondary phases, all compositions with Cs_17_Br_*x*_ show a minor peak corresponding to the nonperovskite δ_Cs_ (Figure S10) due to phase segregation since it overpasses the solubility limit.^12^ It is known that this nonperovskite is photoinactive and unwanted for solar cell applications.^4^

### Br Addition Improves Stability at Low Temperatures

**Figure 4:**
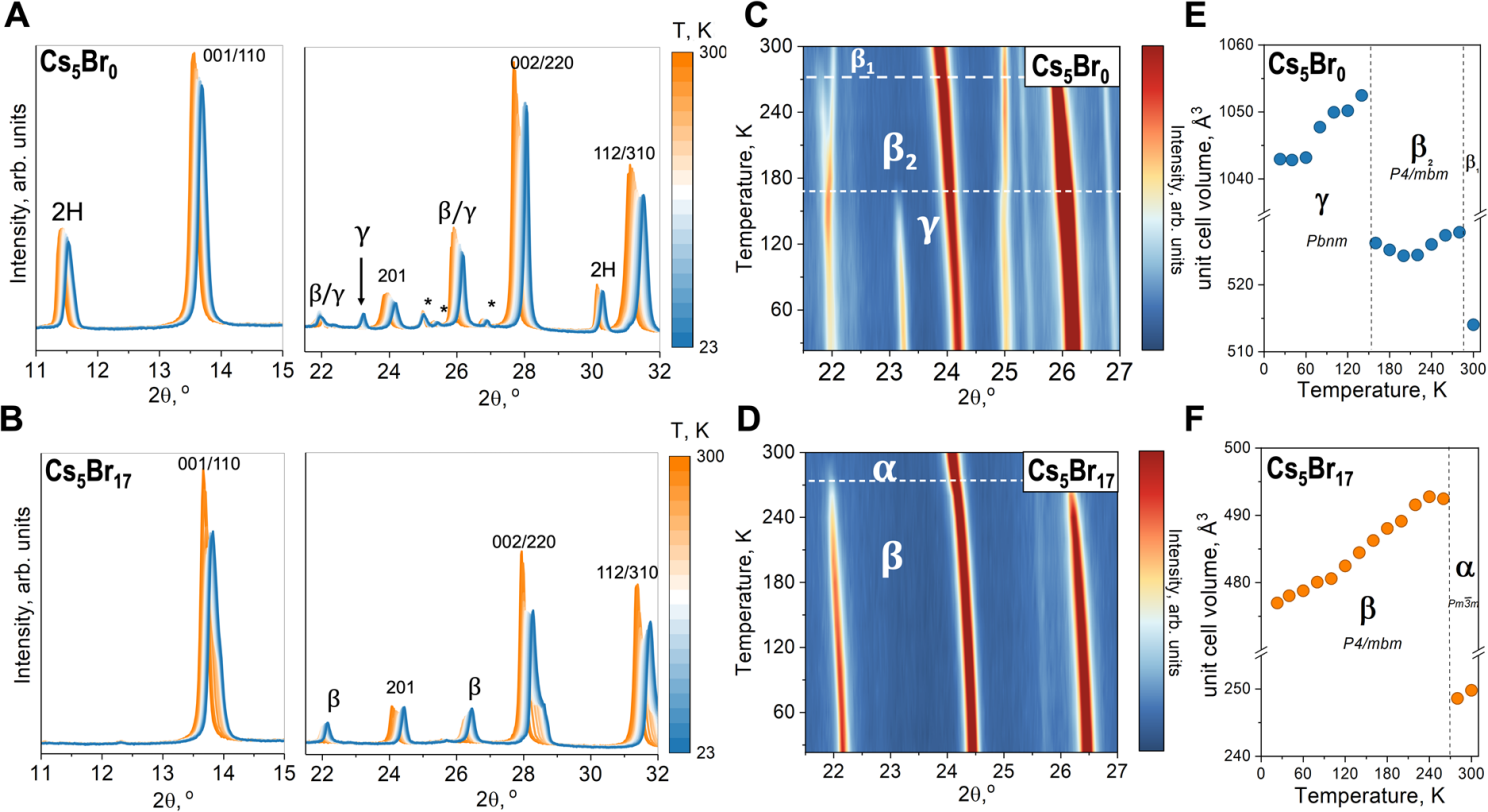
Effect of adding Br into the (Cs,FA)PbI_3_ LHP by in situ XRD in a Cs 5% composition. (A,B) Diffraction patterns for all set of studied temperatures in two ranges of 2θ, from 11 to 15° and 21.5–32° (A) Cs_5_Br_0_ and (B) Cs_5_Br_17_. (C,D) Temperature versus diffraction plot from 21.5 to 27° for (C) Cs_5_Br_0_ and (D) Cs_5_Br_17_. (E,F) Unit cell volume as a function of temperature from Le Bail refinements for (E) Cs_5_Br_0_ and (F) Cs_5_Br_17_.

We evaluated the low-temperature phase transitions for a mixed-cation (Cs,FA) and mixed-halide (I,Br) perovskite, as shown in Figure 1A. In Figure 3, we presented the three phase diagrams to compare different ratios of mixed-cation (Cs,FA) and mixed-halide (I,Br). These phase diagrams reveal that the addition of Br to mixed-cation (Cs,FA) compositions stabilizes the perovskite β phase over a wide temperature range, from approximately 260 K down to 23 K. This stabilization prevents significant structural changes, symmetry variations, and octahedral tilting that occur in the γ phase.^32^Figure 4 illustrates the in situ XRD pattern and results for two compositions, Cs_5_Br_0_ and Cs_5_Br_17_. The complete set of temperature-dependent diffraction patterns can be found in Figures S5–S8. Figure 4A,B shows two significant diffraction regions for the (A) Cs_5_Br_10_ and (B) Cs_5_Br_17_ powders as the samples are cooled from 300 K (orange) to 23 K (blue). The main perovskite Bragg peaks are highlighted, and the Bragg peaks correspond to nonperovskite 2H and δ_Cs_ phases are shown. Figure 4C,D shows the diffraction regions from 21.5 to 27° as a function of temperature for (C) Cs_5_Br_0_ and (D) Cs_5_Br_17_. For Cs_5_Br_0_ in Figure 4C, the perovskite phase starts as β at 300 K, with characteristic peaks around 22 and 26.5° corresponding to the 210 and 211 Bragg peaks of the tetragonal phase. For Cs_5_Br_0_, a new peak appears around 23.2° at 167 K, indicating the presence of the γ phase. In contrast, Figure 4D shows that for Cs_5_Br_17_, the perovskite phase initially adopts the α structure. As the temperature decreases, Bragg peaks of the β phase appear at ∼267 K, and no peak related to the γ phase is observed down to 23 K. Furthermore, Cs_5_Br_17_ does not exhibit any diffraction peaks attributed to secondary phases, maintaining a single perovskite phase structure from 300 to 23 K. For further analysis, from Le Bail refinements, we obtained the unit cell’s lattice parameters (Tables S3 and S4 and Figures S11 and S12) and the unit cell volume (Figures 4E,F). Figure 4E shows the unit cell volume as a function of temperature for a composition without Br, Cs_5_Br_0_. We observed a significant increase in volume from 300 to 280 K. However, in both cases, it fits a tetragonal structure of the space group *P*4/*mbm*. After the initial increase, the volume decreases as temperature decreases, as expected.^9^ Below 160 K, the perovskite phase transforms into the γ phase with space group *Pbnm*. Therefore, the unit cell volume increases and then keeps decreasing as a function of temperature. When Br is added (Cs_5_Br_17_), Figure 4F shows the unit cell volume as a function of temperature, evidencing the phase transition from α to β. For the β phase, there is a linear decrease of the unit cell volume as a function of temperature down to 23 K.

The observed phase transitions and structural behavior in mixed-cation mixed-halide perovskites differ from those reported in single-cation or single-halide systems. Adding Br to single-cation iodide compositions, such as MAPb(I,Br)_3_^9^ or CsPb(I,Br)_3_,^10^ has resulted in a transition from the β to the γ phase. Similarly, in mixed-cation and single-halide systems, such as (Cs,FA)PbI_3_, the transition to the γ phase has been reported in the range of miscibility.^5^ Remarkably, in (Cs,FA)PbBr_3_, Cs substitution has been shown to suppress phase transitions at lower temperatures.^14^ Mozur et al. attributed this phase stability to local compressive strain caused by Cs substitution, overriding the orientation driven by the organic–organic interactions and disrupting changes in low-temperature phase transitions. They highlighted the sensitivity of organic–organic interactions in FAPbBr_3_, explaining the advantages of Cs incorporation in halide perovskites. Other studies have also reported the suppression of phase transitions by incorporating Cs in MAPbBr_3_, attributed to compressive chemical pressure.^32^ Similarly, Ray et al. suppressed the orthorhombic phase of MAPbBr_3_ by adding DMA and forming a mixed-cation (MA,DMA)PbBr_3_ perovskite.^16^ This suppression was attributed to increased disorder of organic cations and a less distorted inorganic framework.^33^ Building upon this understanding, we suggest that the addition of Cs and Br to the FAPbI_3_ perovskite, forming a (Cs,FA)Pb(I,Br)_3_ framework, compresses the structure, as evidenced by smaller lattice parameters, affecting the interactions between organic cations and increasing disorder.

To evaluate the effect of crystal structure variations in the electrical properties of (Cs,FA)Pb(I,Br)_3_ perovskites, we deposited perovskite thin films for 6 selected compositions and used them to fabricate and evaluate the performance of solar cells (Figure S13). In general, by analyzing the crystal structure of the perovskite films using room temperature XRD (Figure S13A), the thin film samples without Cs (Cs_0_Br_*x*_) or Cs_5_Br_*x*_ did not show the same perovskite or nonperovskite phases as the powder samples (Figure 2). We attribute this inconsistency to different thin film deposition parameters, such as solvent and antisolvent, which play a critical role in the crystallization kinetics and phase formation of the metastable perovskite.^34^ Nonperovskite hexagonal phases are detrimental to solar cell performance in FAPbI_3_-based compositions.^35,36^ Therefore, the composition that showed the minimum power conversion efficiency in solar cells was Cs_0_Br_17_ (Figure S13B), partially due to hexagonal secondary phases (Figure S13A). Cs_5_Br_17_ also showed a low-performance solar cell and the presence of nonperovskite phases. The best power conversion efficiency of solar cells was the composition Cs_17_Br_10_, correlated to a single-phase perovskite.

**Figure 5:**
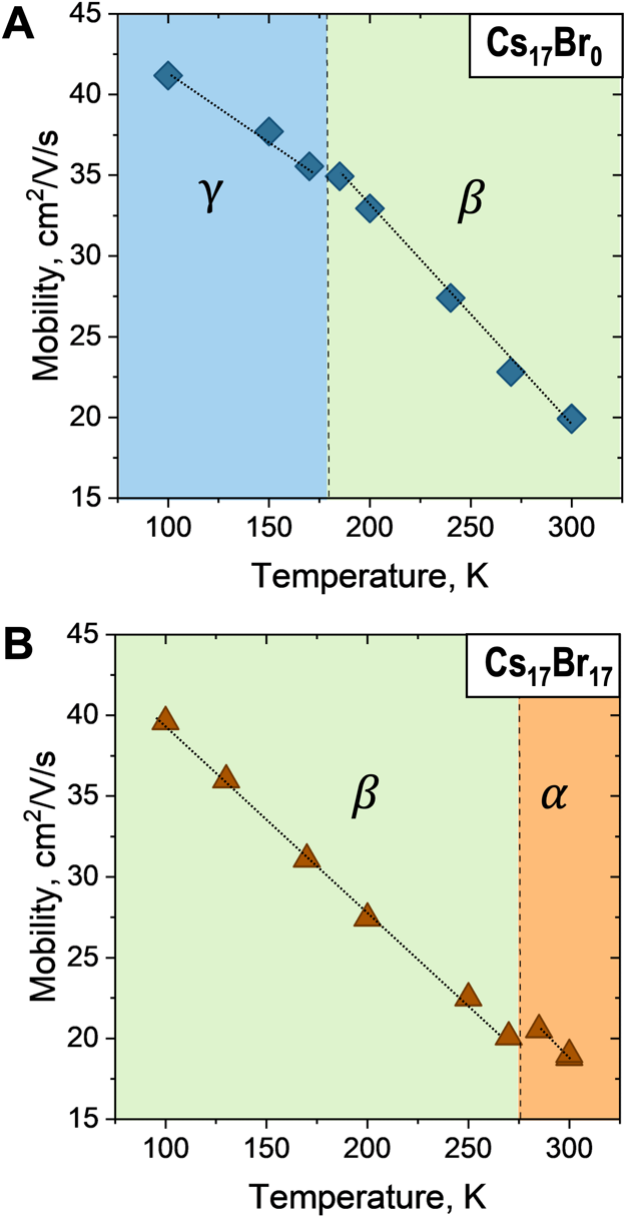
Carrier mobility as a function of temperature. (A) Without Br Cs_17_Br_0_ and (B) with Br Cs_17_Br_17_. Lines are to guide the eye and highlight discontinuity across phase transition temperatures.

To evaluate the charge carrier transport properties, we chose two perovskite thin film compositions with the same crystal structure as their powder samples, one without Br (Cs_17_Br_0_) and one without Cs_17_Br_17_ (Figure S14). We used time-resolved THz spectroscopy to measure the charge carrier mobility as a function of temperature for Cs_17_Br_0_ and Cs_17_Br_17_, as shown in Figure S5 (methods in the Supporting Information). Consistent with literature reports where higher Br content leads to lower carrier mobility, the carrier mobility for Cs_17_Br_17_ is lower than that for Cs_17_Br_0_ at all temperatures.^3,37^ We observed that the carrier mobility increases for both compositions as the temperature decreased from 300 to 100 K. The increase in carrier mobility is expected given that lower phonon vibrations at lower temperatures improve carrier transport.^38^ We observe a small discontinuity in the mobility data, in contrast to other reports where no significant discontinuities across structural phase transitions were observed.^38^ For the film without Br (Cs_17_Br_0_) in Figure 5A, we observe a slope change corresponding to a phase transition at around 175 K, which we attribute to a crystal phase transition from β to γ, close to the powder phase transition temperature in Figures 3C and S8. Similarly, when adding Br (Cs_17_Br_17_), Figure 5B shows the phase transition from α to β at around 270 K, close to the phase transition observed by in situ XRD (Figures 3C and S8). The increase in mobility for both samples at lower temperatures is modest because we average the mobility values across different excited carrier densities (Figures S15 and S16). At higher excitation fluences, carrier–carrier scattering dominates the carrier mobility rather than phonon scattering. By examining the average carrier mobility at both high and low excitation fluences, the impact on carrier transport due to the structural phase transition is visible and not masked by temperature-dependent phonon scattering.^39^ Overall, the carrier mobilities of the two samples across the temperature range probed are very similar, supporting that the crystal symmetry is not the dominant factor in determining carrier transport in perovskite materials.^39^

## Conclusions

In conclusion, this work shows for the first time the low-temperature phase transitions for a mixed-cation and mixed-halide perovskite. We measured in situ powder XRD to create temperature phase diagrams for different concentrations of Cs and Br in (Cs,FA)Pb(I,Br)_3_ perovskites. By replacing I with Br in (Cs,FA) compositions, we found that the transition from a tetragonal to orthorhombic phase at low temperatures (below 150 K) was suppressed, resulting in a more symmetrical perovskite structure with potential applications in low-temperature settings. When Cs was not present, we observed the formation of nonperovskite hexagonal phases. However, the addition of Cs and Br prevented the formation of hexagonal structures. It is important to note that the inclusion of 17% Cs resulted in the formation of the secondary nonperovskite orthorhombic δ-CsPbI_3_ phase. From these findings, it can be concluded that adding Br to (Cs,FA) perovskites enhances the stability of the β phase over a wider temperature range, which is advantageous for low-temperature applications. Although the introduction of Br increases the bandgap of the perovskite, which reduces the number of absorbed photons, the overall crystal structure becomes more robust and contributes to long-term stability. To gain a comprehensive understanding of how the crystal structure behaves across the entire temperature range, further investigations focusing on the fundamental structural aspects are essential.

## Methodology

### Experimental Section

#### Mixed-Cation Mixed-Halide (Cs,FA)Pb(I,Br)_3_ Precursor Solution

Mixed-cation and mixed-halide Cs_*y*_FA_1–*y*_Pb(I_*x*_Br_1–*x*_)_3_ perovskites were prepared with different molar ratios of Cs/FA (*y*) and Br/I (*x*). The precursor powders used were CsI (Sigma), FAI (TCI), PbI_2_ (TCI), and PbBr_2_ (TCI). The precursor powders were weighted on the same vial to form a 0.5 M solution in a mixed solvent of gamma-butyrolactone (GBL) and dimethylformamide (DMF) with a volume ratio of 2:1. The precursor solution was mixed at 600 rpm and 60 °C for 1 h. The solutions were stoichiometric A/Pb (1:1).

#### Powder Synthesis

After the precursor solution was mixed, it was evaporated at 170 °C for 40 min on a glass Petri dish. A solid was formed on the Petri dish, which was broken into pieces with a spatula and left sintering on the hot plate at 170 °C for 1 h. Afterward, this solid was completely removed and ground into a very fine powder with a mortar.

### Characterization

#### UV–vis

We measured UV–vis absorption spectroscopy using the PerkinElmer UV/vis, NIR Spectrometer Lambda 750 S, at the Helmholtz Zentrum Berlin (HZB). The spectrometer has a double-beam, double-monochromator, ratio-recording optical system. The instrument has a Praying Mantis Diffuse Reflection accessory in the sample compartment and a 100 mm integrating sphere accessory in the detector. This instrument is suitable for the measurement of powders.

#### In Situ Powder XRD

For the set of powder LHPs mixed-cations and mixed-halides, the temperature-dependent phase transitions were measured by in situ XRD. Synchrotron-based in situ XRD was measured at beamline KMC-2 in BESSY II at the HZB, Germany. The instrument used a Bruker Vantec 2000 area detector. The X-rays had an energy of 8 keV optimized for the beamline. For the temperature controller, a closed cycle refrigerator of the reference TMP-CCR-HXR was used. XRD patterns were taken from 300 to 20 K in steps of 20 K. This beamline offered both the experimental precision as well as the sample environment to perform such investigations. Powder studies were also important for a better indication of the thermodynamic processes due to a more pronounced crystallization process, less exposed surfaces, a larger probed volume compared to thin film samples, and better instrumental resolution. Le Bail fittings using the FullProf software were done to calculate the lattice parameters and phase refinement for the samples at 300 K.

#### Temperature-Dependent Carrier Mobility

Charge carrier transport was characterized by using time-resolved THz spectroscopy. Details of the setup can be found in,^40^ with the important parameters described briefly here. The perovskite samples deposited on double-side-polished z-cut quartz substrates were excited by a femtosecond laser pulse with the wavelength centered at 400 nm, focusing to an area with a diameter of 2 mm. The carrier transport properties were measured using a subpicosecond THz pulse focused onto the center 1 mm diameter spot of the excited area. The THz transmission amplitude was recorded using an electro-optic sampling technique and a lock-in amplifier.^41^ Free mobile charge carriers attenuate THz probe pulses, and the transmission reduction was proportional to the photoconductivity of the sample. The samples were loaded into a continuous-flow cryostat (Janice ST-100H) with z-cut quartz windows for transparency in the THz frequency range. Samples were loaded in the vacuum chamber and cooled to liquid nitrogen temperature first, and the measurements were conducted on the heating cycle between 100 and 300 K. At each temperature, samples were excited at three excitation densities, corresponding to laser fluences of 30, 90, and 140 μJ/cm^2^. After excitation at the highest laser fluence, repeated measurements at a low fluence ensured that there was no sample degradation. Room temperature measurements were conducted before the temperature cycle for comparison to the room temperature measurement at the end of the experiment to ensure that no sample degradation occurred during the measurement. The reported carrier mobility was the average mobility measured with the three excited fluences.

## Supporting Information Available

UV–vis spectra; Le Bail refinements and lattice parameters at 300 K; complete XRD patterns for all compositions at all temperatures; in situ temperature XRD 2D maps for all compositions; phase transformation temperatures for all compositions; lattice parameter values for volume calculations; perovskite thin films XRD; solar cell measurements and statistics; thin films XRD used for THz conductivity measurements; and raw transient THz conductivity data (PDF)
