# Supplementary material for: Br-Induced Suppression of Low-Temperature Phase Transitions in Mixed-Cation Mixed-Halide Perovskites
Source: Chem Mater. 2024 Oct 4;36(20):10167–75. doi: 10.1021/acs.chemmater.4c01670 (PMC11500282; doi:10.1021/acs.chemmater.4c01670)
Supplement: Supplementary file 1 — cm4c01670_si_001.pdf [file cm4c01670_si_001.pdf]

Supporting Information

Br-Induced Suppression of Low-Temperature Phase Transitions in Mixed Cation Mixed Halide Perovskites

Juanita Hidalgo[1,2], Joachim Breternitz[2], Daniel M. Többens[2], Diana K. LaFollette[1], Charles N.B. Pedorella[3], Meng-Ju Sher[3], Susan Schorr[2,4*], Juan-Pablo Correa-Baena[1*]

[1] School of Materials Science and Engineering, Georgia Institute of Technology, Atlanta, Georgia 30332, United States.

[2] Department Structure and Dynamics of Energy Materials, Helmholtz-Zentrum Berlin für Materialien und Energie, Hahn-Meitner-Platz 1, 14109, Berlin, Germany.

[3] Department of Physics, Wesleyan University, Middletown, CT 06459, United States.

[4] Freie Universitaet Berlin, Institute of Geological Sciences, Malteser Str. 74-200, 12249 Berlin, Germany.

*Corresponding Authors: Juan-Pablo Correa-Baena jpcorrea@gatech.edu and Susan Schorr susan.schorr@helmholtz-berlin.de

1. UV-VIS Absorption

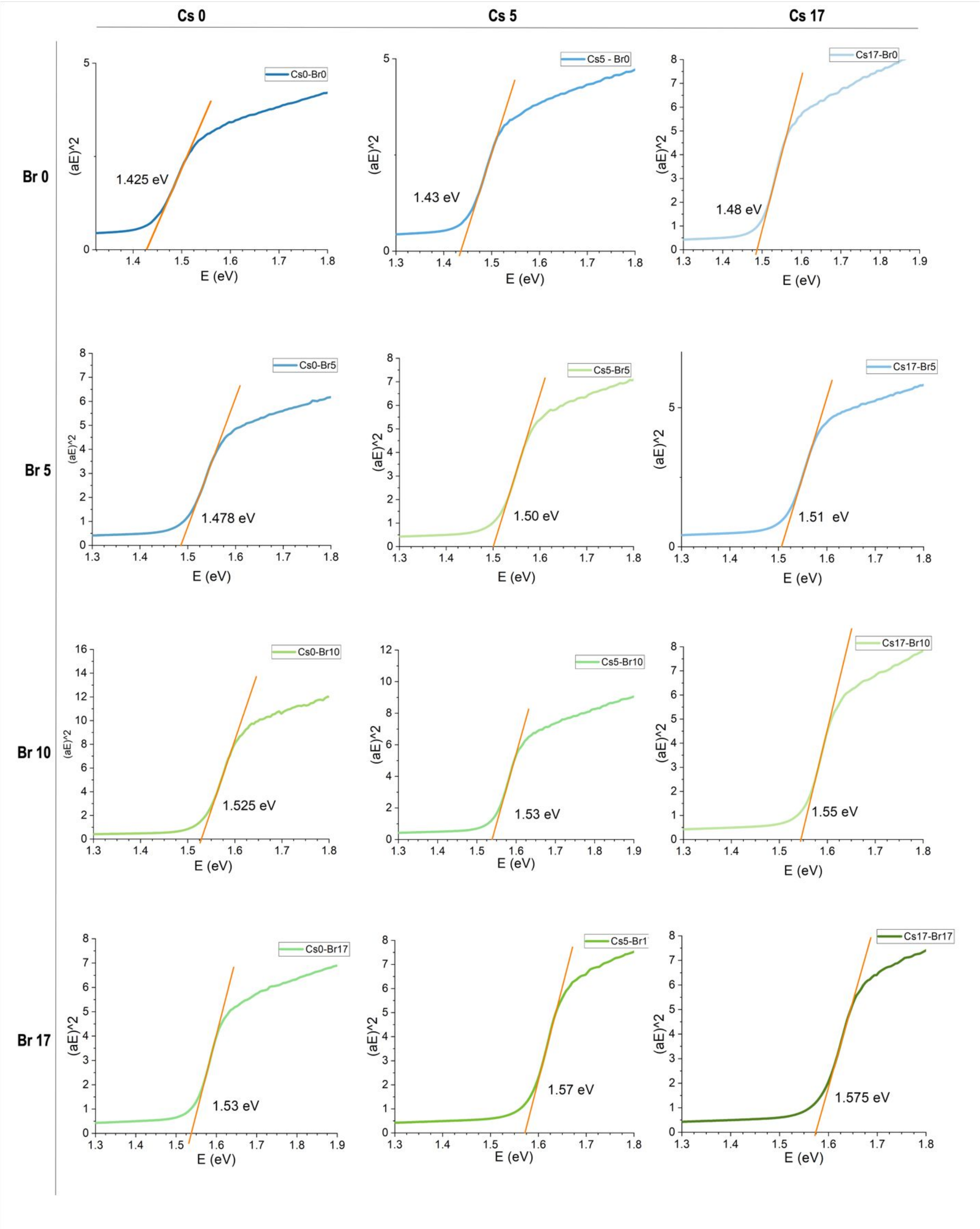

Figure S1. Tauc-plots from UV-VIS absorption for the set of studied compositions.

2. LeBail Refinements at T = 300 K

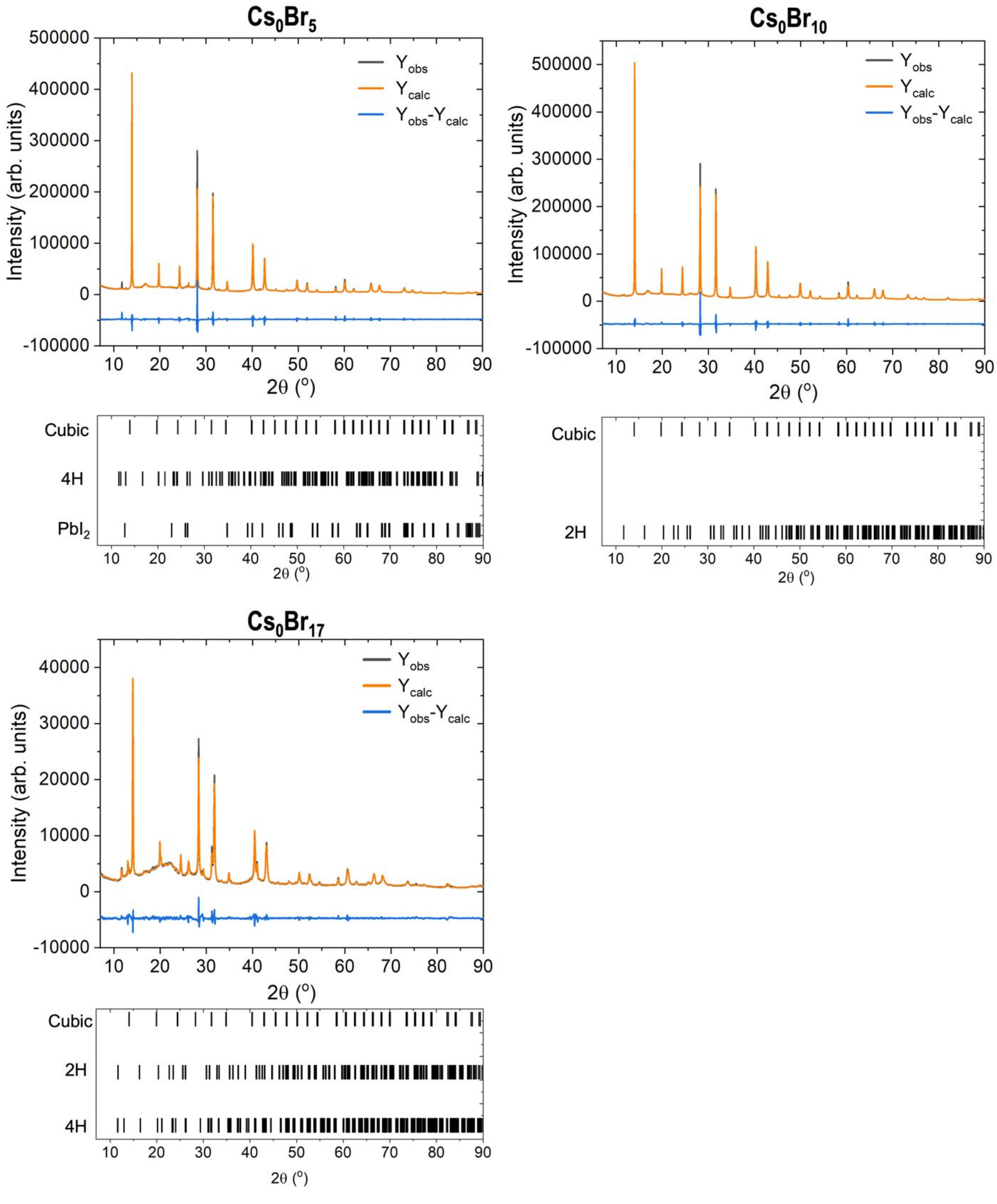

Figure S2. Le Bail refinements from powder XRD at 300 K, compositions Cs 0 % and Br variations

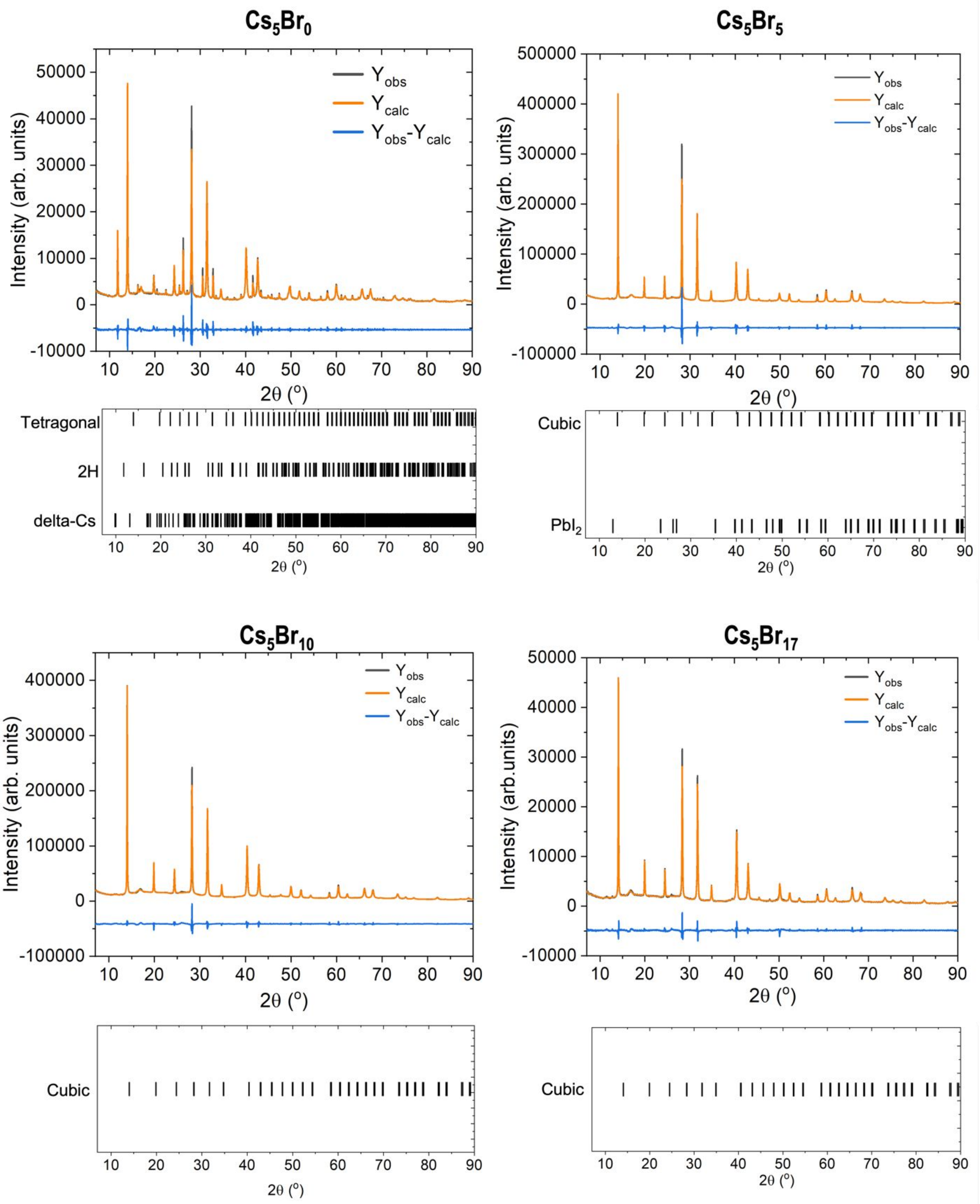

Figure S3. Le Bail refinements from powder XRD at 300 K, compositions Cs 5 % and Br variations.

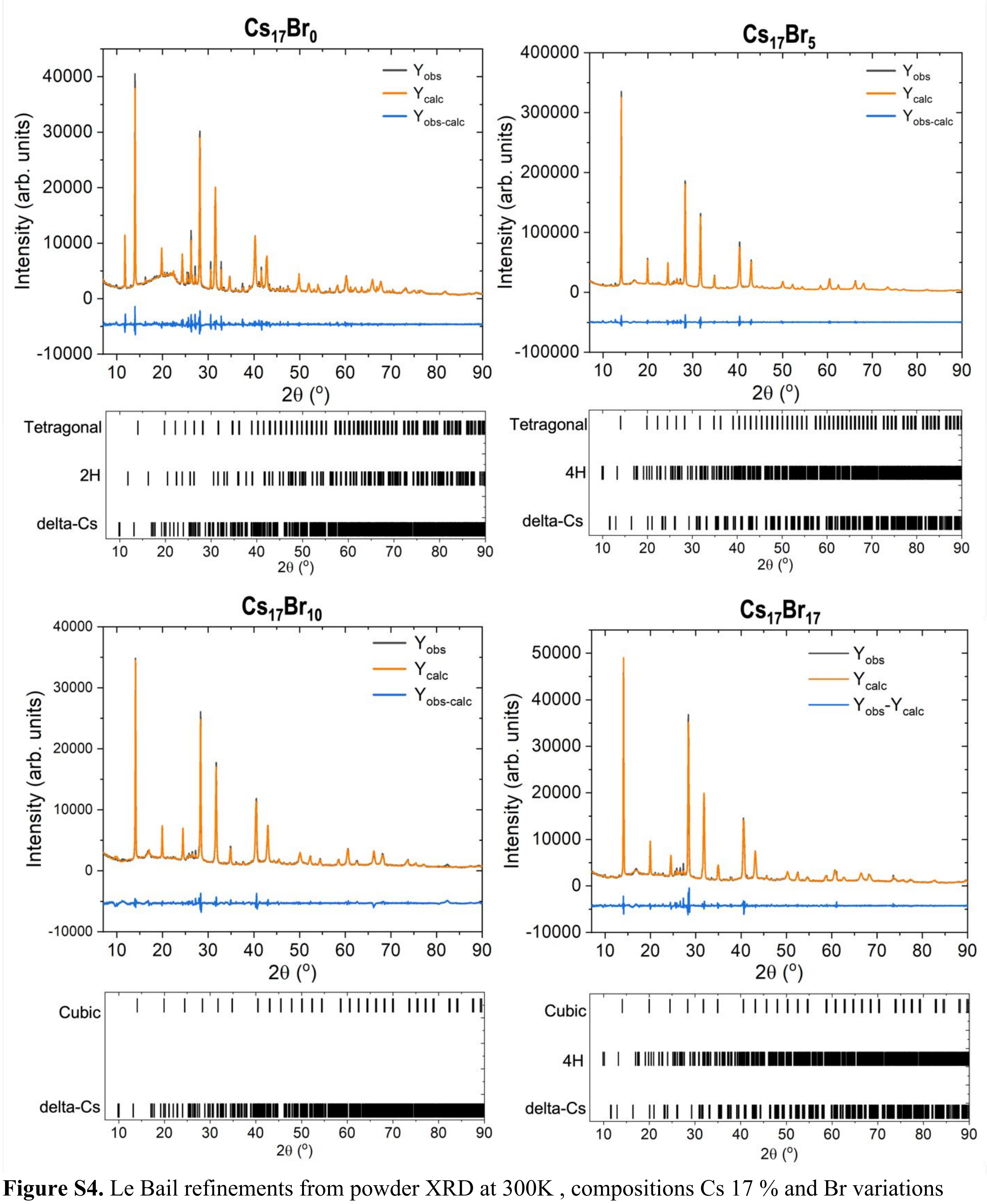

Figure S4. Le Bail refinements from powder XRD at 300K , compositions Cs 17 % and Br variations

Table S1. Lattice parameters from Le Bail refinements for compositions at 300 K

				Lattice Parameters					
	**Br**	**Phase**	**Space group**	**a (Å)**	**b(Å)**	**c (Å)**	**alpha(°)**	**beta(°)**	**gamma(°)**
**Cs0**	5	cubic	$Pm\bar{3}m$	6.35(0)	6.35(0)	6.35(0)	90	90	90
		4H	$P6_3/mmc$	8.81(6)	8.81(6)	14.73(0)	90	90	120
		PbI_2	$P\bar{3}m1$	4.476(0)	4.476(0)	6.87(5)	90	90	120
	10	cubic	$Pm\bar{3}m$	6.33(1)	6.33(1)	6.33(1)	90	90	90
		2H	$P6_3/mmc$	8.72(8)	8.72(8)	7.85(0)	90	90	120
	17	cubic	$Pm\bar{3}m$	6.30(6)	6.30(6)	6.30(6)	90	90	90
		2H	$P6_3/mmc$	8.73(1)	8.73(1)		90	90	120
		4H	$P6_3/mmc$	8.80(7)	8.80(7)	15.16(6)	90	90	120
**Cs5**	0	tetragonal	$P4/mbm$	8.99(1)	8.99(1)	6.35(9)	90	90	90
		2H	$P6_3/mmc$	8.68(1)	8.68(1)	7.93(2)	90	90	120
		delta Cs	*Pnma*	10.46(7)	4.79(4)	17.7(1)	90	90	90
	5	cubic	$Pm\bar{3}m$	6.33(8)	6.33(8)	6.33(8)	90	90	90
		PbI_2	$P\bar{3}m1$	4.38(5)	4.38(5)	6.81(8)	90	90	120
	10	cubic	$Pm\bar{3}m$	6.31(9)	6.31(9)	6.31(9)	90	90	90
	17	cubic	$Pm\bar{3}m$	6.29(8)	6.29(8)	6.29(8)	90	90	90
**Cs17**	0	tetragonal	$P4/mbm$	8.97(3)	8.97(3)	6.32(7)	90	90	90
		2H	$P6_3/mmc$	8.67(7)	8.67(7)	7.93(4)	90	90	120
		delta Cs	*Pnma*	10.45(8)	4.80(3)	17.68(2)	90	90	90
	5	tetragonal	$P4/mbm$	8.92(6)	8.92(6)	6.31(6)	90	90	90
		4H	$P6_3/mmc$	8.83(9)	8.83(9)	15.19(0)	90	90	120
		delta Cs	*Pnma*	10.42(6)	4.81(4)	17.41(0)	90	90	90
	10	cubic	$Pm\bar{3}m$	6.30(8)	6.30(8)	6.30(8)	90	90	90
		delta Cs	*Pnma*	10.41(0)	4.80(0)	17.6(0)	90	90	90
	17	cubic	$Pm\bar{3}m$	6.28(7)	6.28(7)	6.28(7)	90	90	90
		4H	$P6_3/mmc$	8.83(9)	8.83(9)	15.19(0)	90	90	120
		delta Cs	*Pnma*	10.43(5)	4.81(5)	17.40(8)	90	90	90

3. *In-situ* low-temperature powder XRD and phase transition temperatures

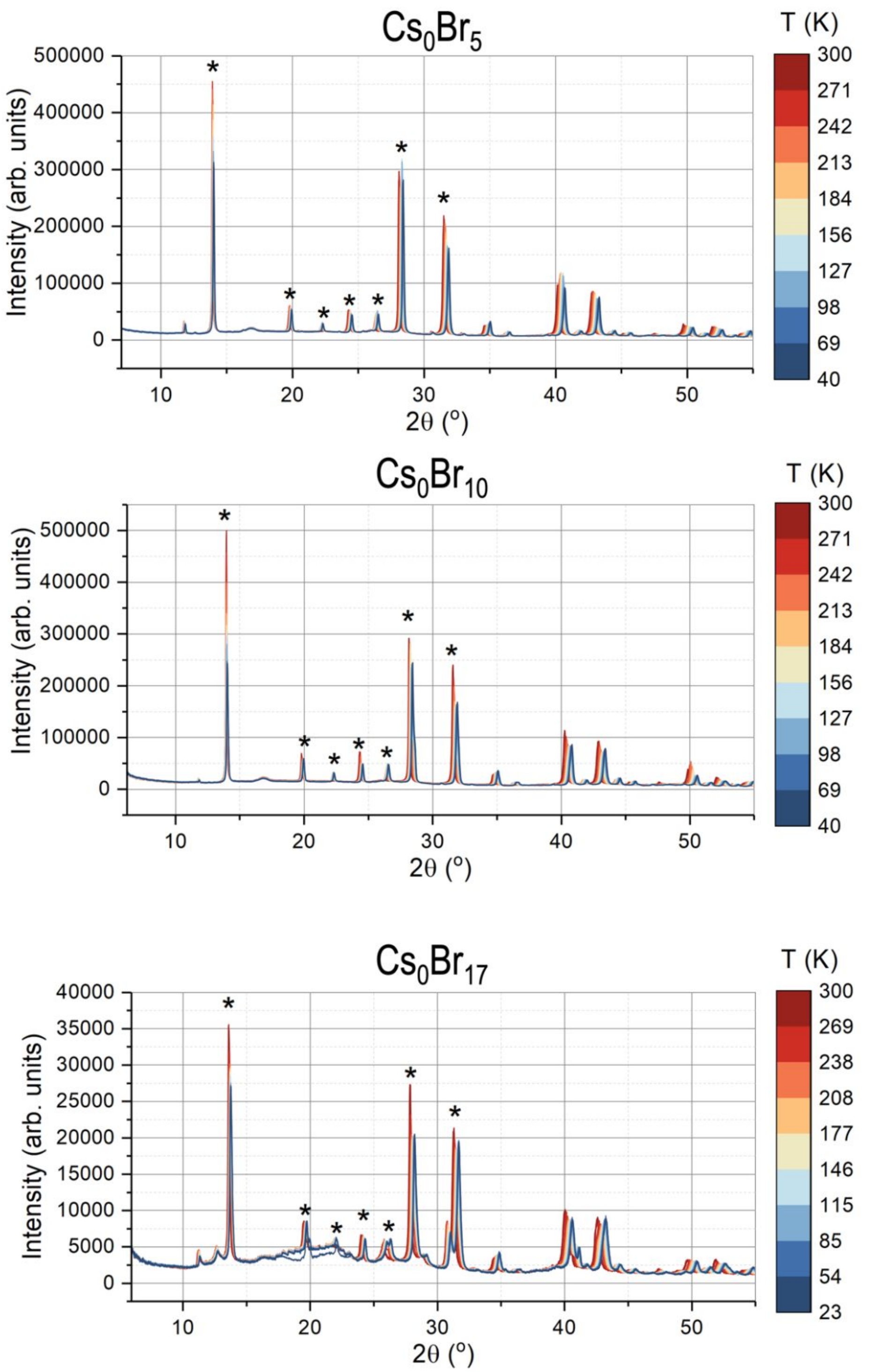

Figure S5. Complete set of *in-situ* XRD from 300 K to 23 K, Cs 0 % and Br variations. The perovskite phases are marked with *, other peaks are from non-perovskite secondary phases.

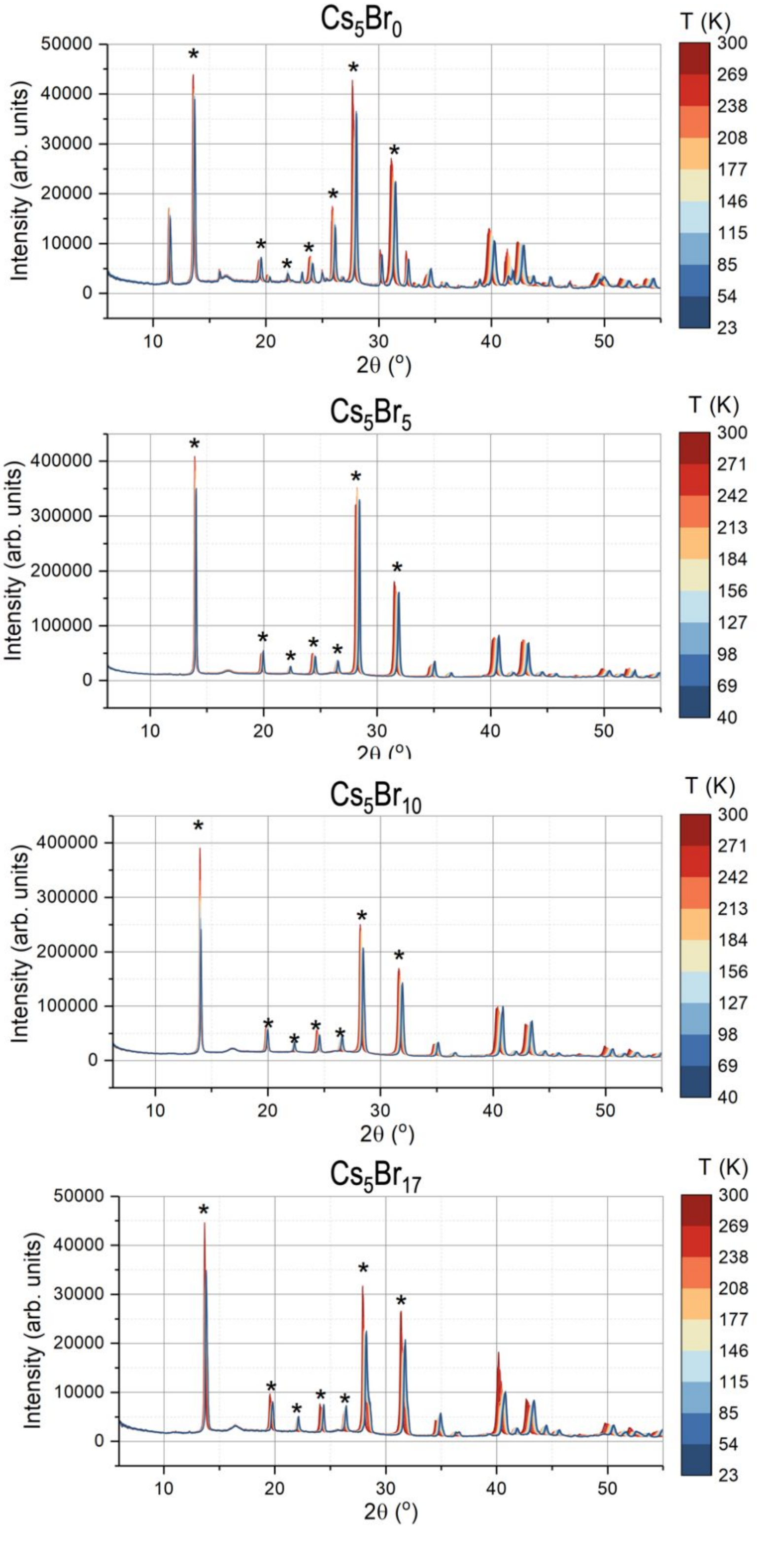

Figure S6. Complete set of *in-situ* XRD from 300 K to 23 K, Cs 5 % and Br variations. The perovskite phases are marked with *, other peaks are from non-perovskite secondary phases.

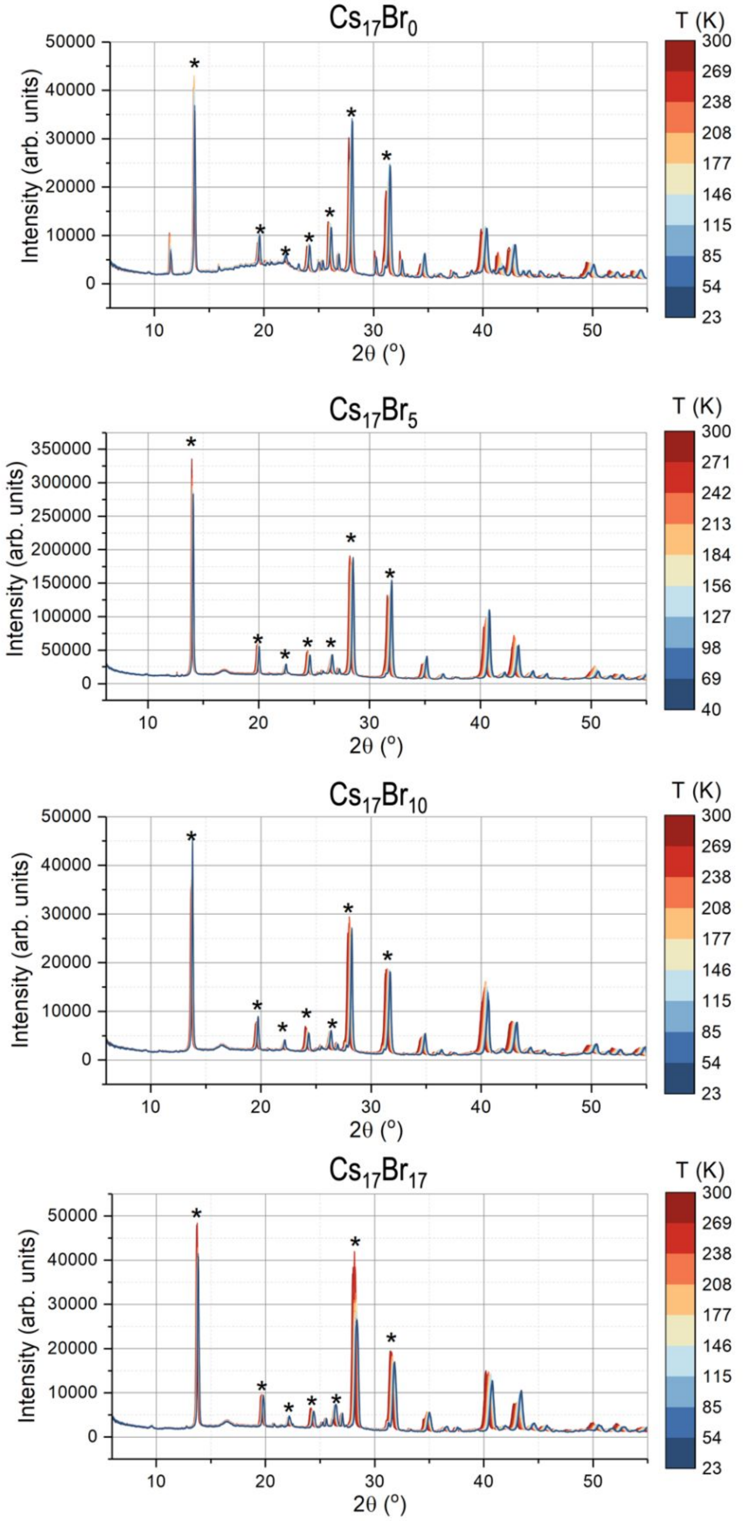

Figure S7. Complete set of *in-situ* XRD from 300 K to 23 K, Cs 17 % and Br variations. The perovskite phases are marked with *, other peaks are from non-perovskite secondary phases.

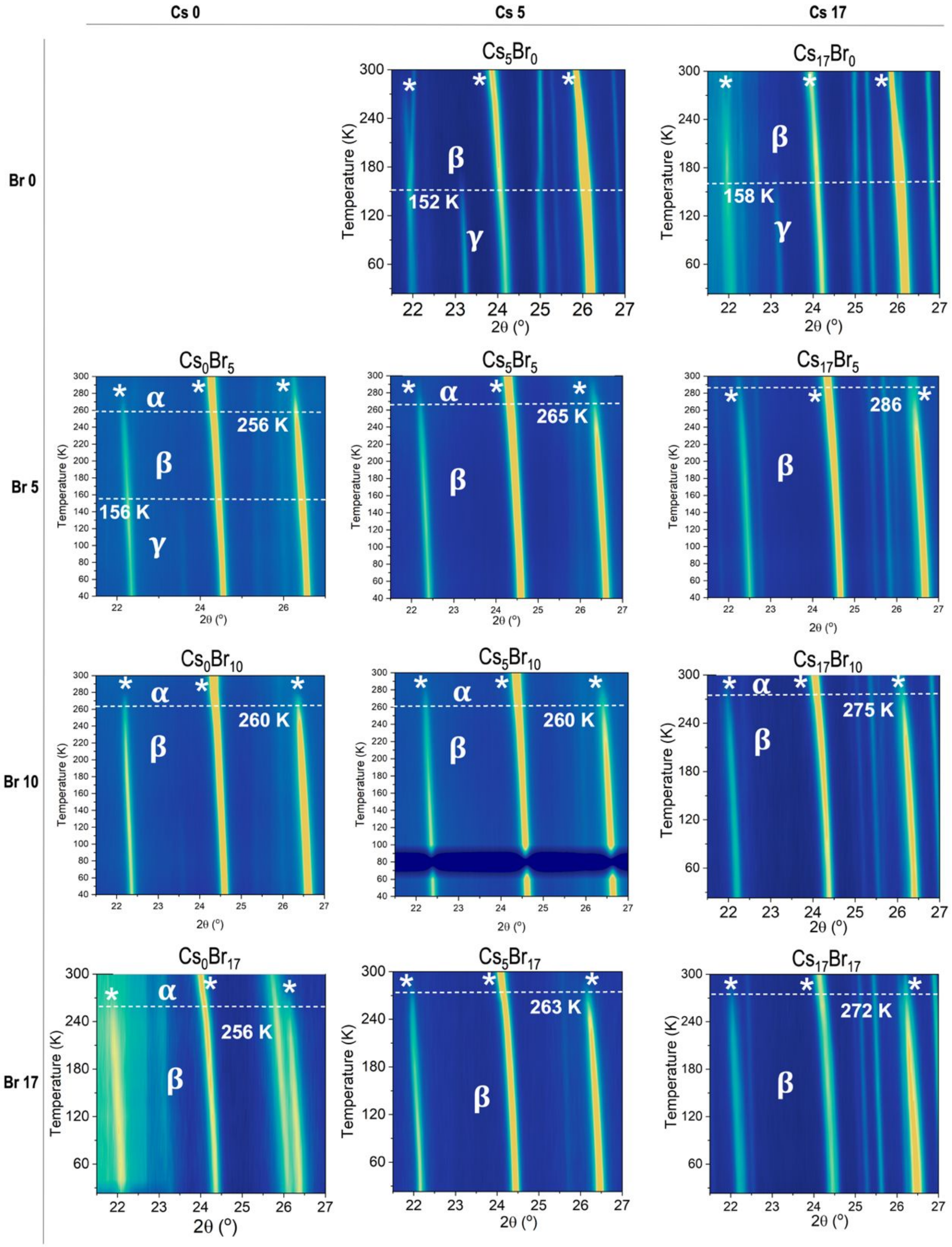

Figure S8. Temperature versus 2q plots (q is the diffraction angle), in the range of phase transitions for all Cs-Br compositions. The perovskite phases are marked with *, other peaks are from non-perovskite secondary phases.

Note on in-situ XRD: The data for composition Cs5Br10 at 80 K was not saved.

Table S2. Temperature phase transitions

		Temperature (K)	
	**Br**	cubic-tetragonal	tetragonal-orthorhombic
**Cs 0**	5	256	156
	10	260	-
	17	256	-
**Cs 5**	0	>300	152
	5	265	-
	10	260	-
	17	263	-
**Cs 17**	0	>300	158
	5	286	-
	10	275	-
	17	272	-

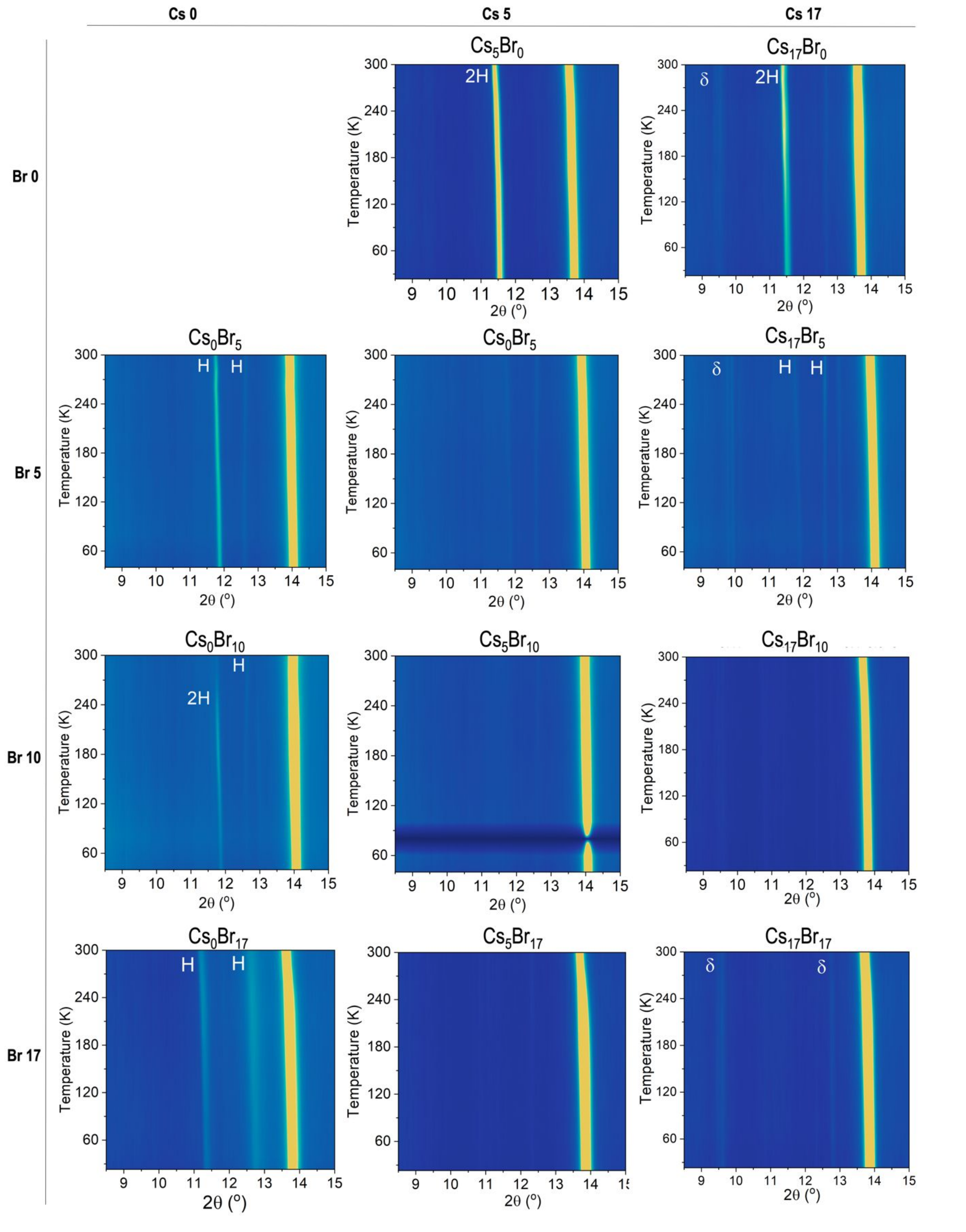

Figure S9. Temperature versus 2q plots (q is the diffraction angle), in the range of hexagonal phases for all Cs-Br compositions. The hexagonal phases are labeled with H.

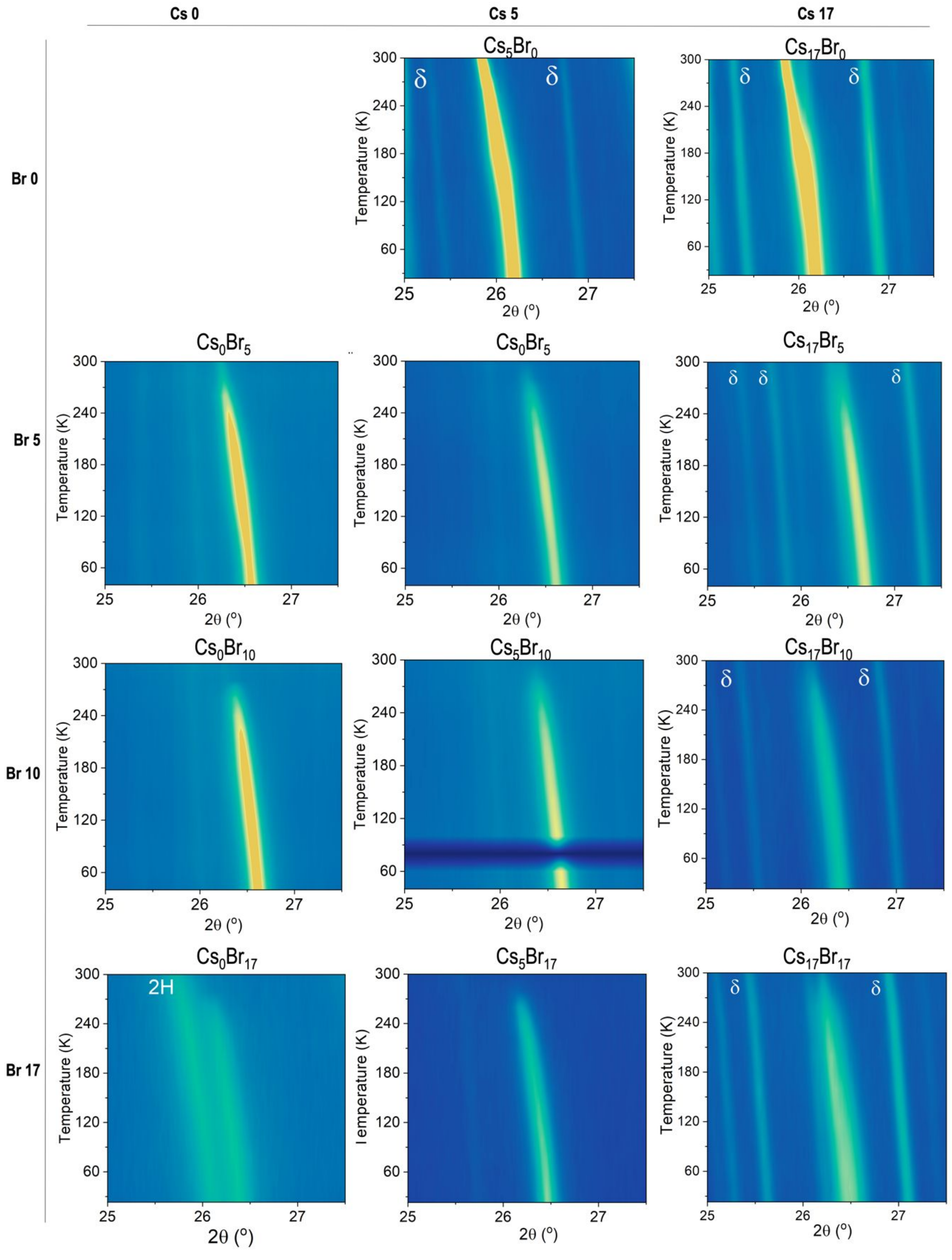

Figure S10. Temperature versus 2q plots (q is the diffraction angle), in the range of hexagonal phases for all Cs-Br compositions. The orthorhombic δ-$CsPbI_3$ is labeled as δ.

4. Temperature vs. unit cell volume plots

To analyze the phase transitions without and with Br, we did Le Bail refinements to all XRD patterns for Cs_5Br_0 and Cs_5Br_{17}, and all temperatures (300 K to 23 K, in 20 K steps). Tables S3 and S4 shows the obtained lattice parameters for the respective space group, and the calculated unit cell volume.

Table S3. Lattice Parameters from Le Bail refinements for $Cs_5FA_{95}PbI_3$

**Cs_5 Br_0**					
**T/ K**	**space group**	**a/ Å**	**b/ Å**	**c/ Å**	**vol/Å^3**
300	*P4/mbm*	8.99(1)	8.99(1)	6.35(9)	514.0
280	*P4/mbm*	9.10(5)	9.10(5)	6.36(7)	527.8
260	*P4/mbm*	9.10(3)	9.10(3)	6.36(4)	527.4
240	*P4/mbm*	9.09(7)	9.09(7)	6.35(6)	526.0
220	*P4/mbm*	9.08(8)	9.08(8)	6.35(0)	524.4
200	*P4/mbm*	9.08(9)	9.08(9)	6.34(7)	524.3
180	*P4/mbm*	9.09(7)	9.09(7)	6.34(6)	525.2
160	*P4/mbm*	9.10(7)	9.10(7)	6.34(5)	526.2
140	*Pbnma*	9.17(4)	9.03(1)	12.70(3)	1052.5
120	*Pbnma*	9.16(8)	9.02(3)	12.69(5)	1050.2
100	*Pbnma*	9.17(0)	9.02(2)	12.69(1)	1049.9
80	*Pbnma*	9.17(1)	9.00(9)	12.68(0)	1047.7
60	*Pbnma*	9.16(0)	8.99(3)	12.66(3)	1043.2
40	*Pbnma*	9.16(0)	8.99(2)	12.66(0)	1042.8
20	*Pbnma*	9.16(3)	8.99(2)	12.65(8)	1043.0

Cs_5Br_0, T = 300 K

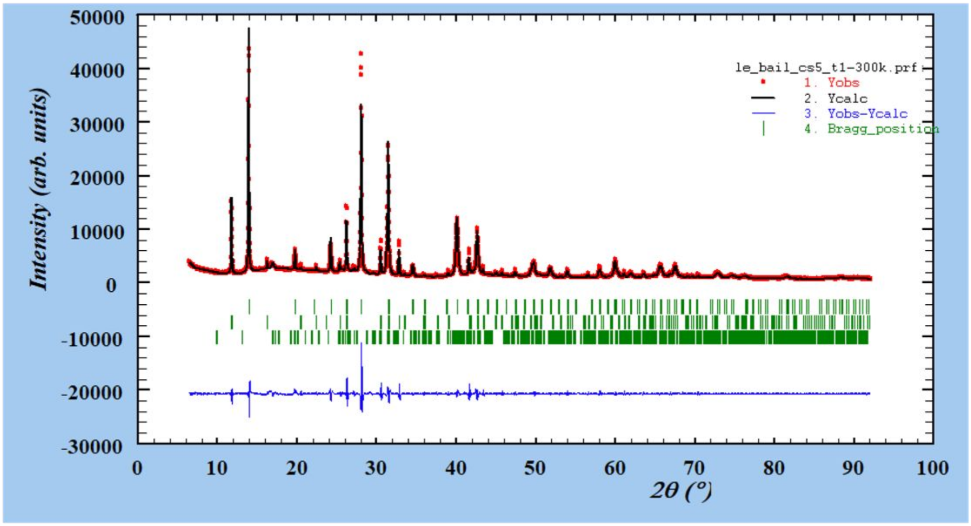

Cs_5Br_0, T = 20 K

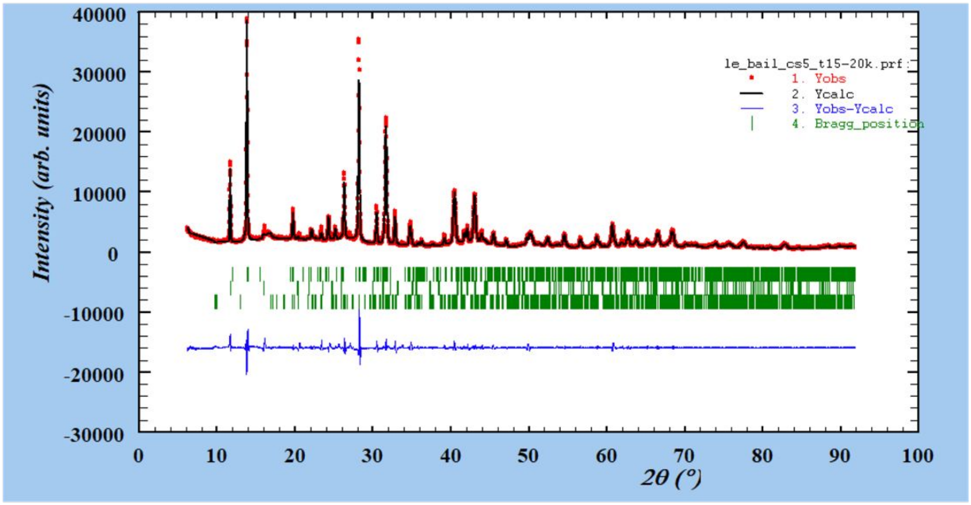

Figure S11. Example of LeBail plots for $Cs_5FA_{95}PbI_3$ at the extreme temperatures (300 K and 20 K). The Bragg positions correspond to three phases: the perovskite, the hexagonal 2H-FAPbI3 and orthorhombic δ-$CsPbI_3$

Table S4. Lattice Parameters from Le Bail refinements for $Cs_5FA_{95}Pb(Br_{17}I_{83})_3$

Cs_5 Br_{17}					
**T/ K**	**space group**	**a/ Å**	**b/ Å**	**c/ Å**	**vol/ Å³**
300	*Pm3m*	6.29(8)	6.29(8)	6.29(8)	249.8
280	*Pm3m*	6.28(8)	6.28(8)	6.28(8)	248.6
260	*P4/mbm*	8.86(8)	8.86(8)	6.26(2)	492.5
240	*P4/mbm*	8.87(6)	8.87(6)	6.25(5)	492.8
220	*P4/mbm*	8.88(8)	8.88(8)	6.22(2)	491.5
200	*P4/mbm*	8.87(6)	8.87(6)	6.20(9)	489.1
180	*P4/mbm*	8.87(0)	8.87(0)	6.20(3)	488.0
160	*P4/mbm*	8.85(8)	8.85(8)	6.19(7)	486.3
140	*P4/mbm*	8.84(7)	8.84(7)	6.18(9)	484.5
120	*P4/mbm*	8.83(2)	8.83(2)	6.18(5)	482.5
100	*P4/mbm*	8.82(0)	8.82(0)	6.17(8)	480.6
80	*P4/mbm*	8.81(6)	8.81(6)	6.17(7)	480.1
60	*P4/mbm*	8.80(8)	8.80(8)	6.17(2)	478.8
40	*P4/mbm*	8.80(2)	8.80(2)	6.17(0)	478.0
20	*P4/mbm*	8.79(3)	8.79(3)	6.16(9)	477.0

Cs_5Br_{17}, T = 300 K

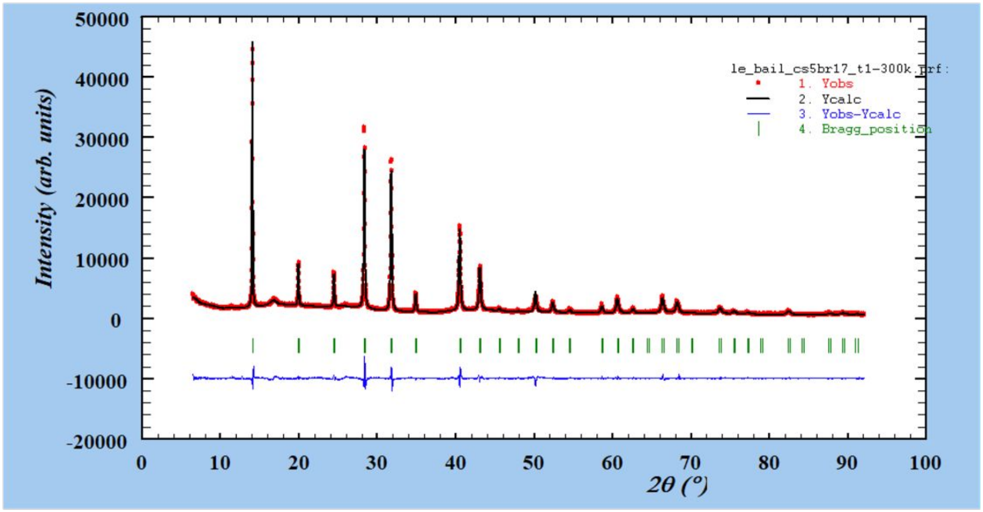

Cs_5Br_{17}, T = 20 K

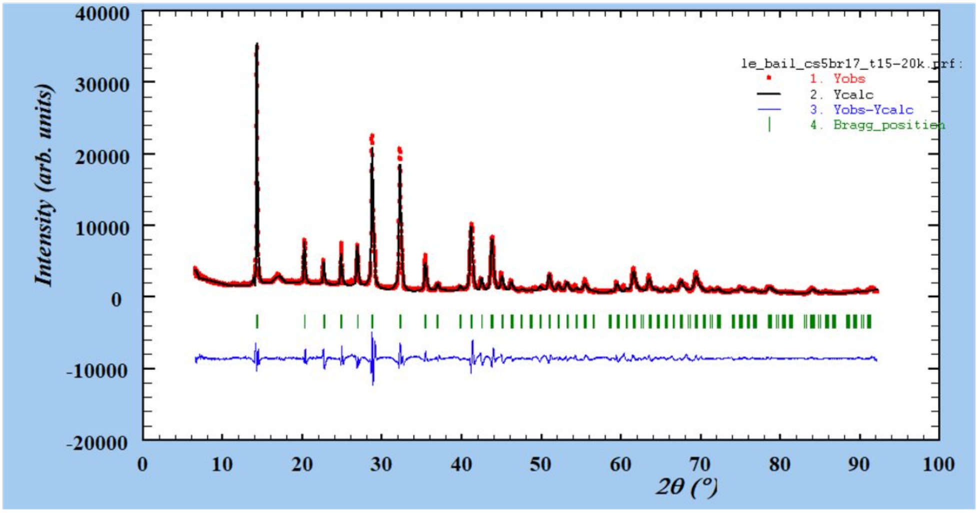

Figure S12. Example of LeBail plots for $Cs_5Br_{17}FA_{95}PbI_3$ at the extreme temperatures (300 K and 20 K). The Bragg positions correspond to one phases: perovskite.

5. Perovskite Solar Cells

The solar cells were fabricated following the procedure published by Saliba et al.[1] for a n-i-p solar cell.

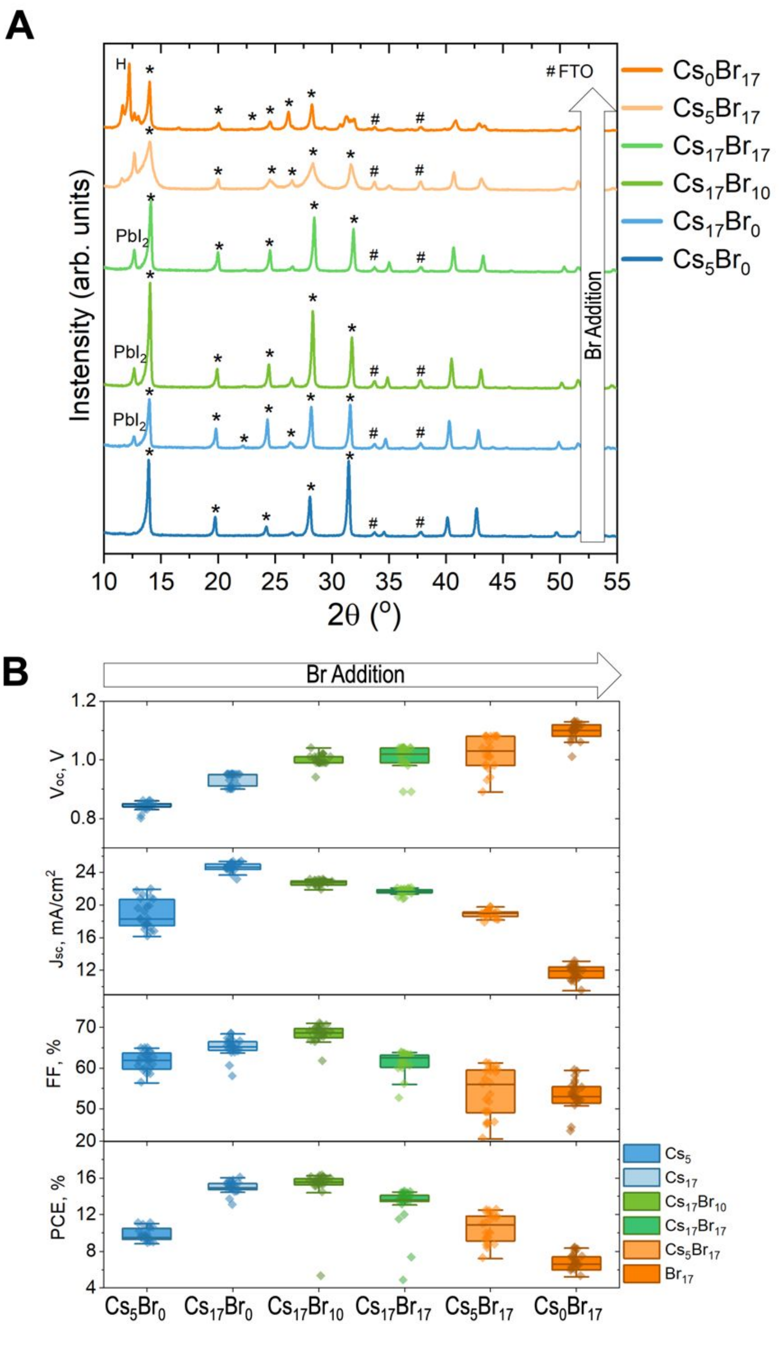

Figure S13. (Cs,FA)Pb(I,Br)$_3$ thin films and solar cells. (A) Boxplot of figures of merit of perovskite solar cells: open circuit voltage (V_{oc}), short circuit current (J_{sc}), fill factor (FF), and power conversion efficiency (PCE). **(B)** XRD pattern from these perovskite thin films on FTO.

6. Temperature-dependent charge mobility in thin films

To evaluate the effect of structure and Br addition on charge carrier mobility, we fabricated thin films of two perovskite compositions: one without Br and one with Br both Cs 17 % given the phase stability of this composition in thin films, as shown in Figure S13A. We repeated XRD on the thin films used in the THz spectroscopy measurements on quartz substrates. We confirm that at room temperature, the $Cs_{17}Br_0$ shows the tetragonal phase Bragg peaks (* in Figure S14), and $Cs_{17}Br_{17}$ has a cubic phase.

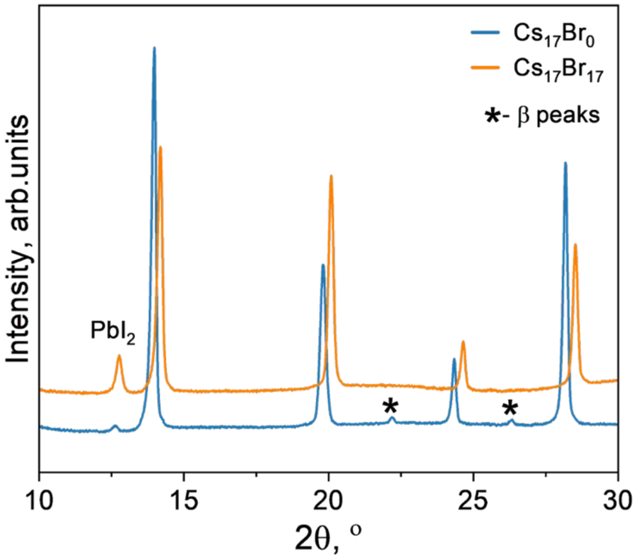

Figure S14. XRD of thin films at room temperature and ambient atmosphere used in THz spectroscopy measurements.

Figures S15 and S16 show the raw THz transient conductivity data for $Cs_{17}Br_0$ and $Cs_{17}Br_{17}$, respectively. At each temperature setting, we collected THz transient photoconductivity data at three laser excitation fluences, 30, 90, and 140 μJ/cm^2, shown in red, black, and blue traces, respectively. The carrier mobility is determined by the change in THz transmission amplitude at t=0 when the THz probe pulse overlaps in time with the 400-nm photoexcitation pulse. We assume that each incident photon generates a free charge carrier and calculate the mobility from $\Delta\sigma = q\mu\Delta n$, where $\Delta\sigma$ is the photoconductivity, q is the elementary charge, μ is the mobility, and Δn is the fluence-dependent excited carrier density. The photoconductivity $\Delta\sigma$ relates to the THz transmission amplitude change by $\Delta\sigma = \frac{1+N_{quartz}}{Z_0 d}\frac{-\Delta T/T_0}{1+\Delta T/T_0}$, where ΔT is the THz transmission amplitude, T_0 is the initial THz transmission through the sample, Z_0 = 377 Ω is the impedance of air, d is the absorption depth, and N_{quartz} is the refractive index of the quartz substrate at THz frequencies. The mobility reported in this manuscript is the mobility averaged over all three excitation fluences.

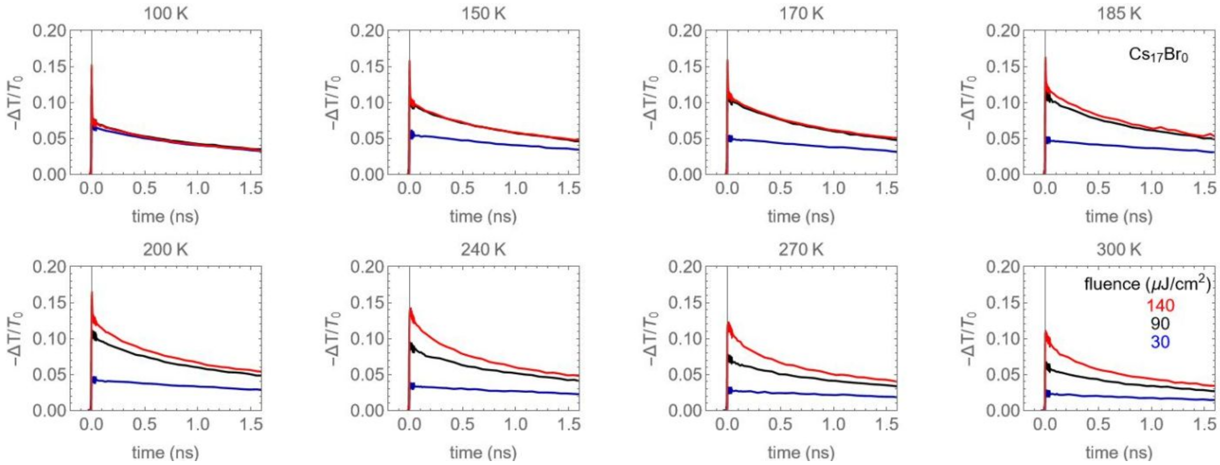

Figure S15. Raw transient THz conductivity data for the $Cs_{17}Br_0$ sample.

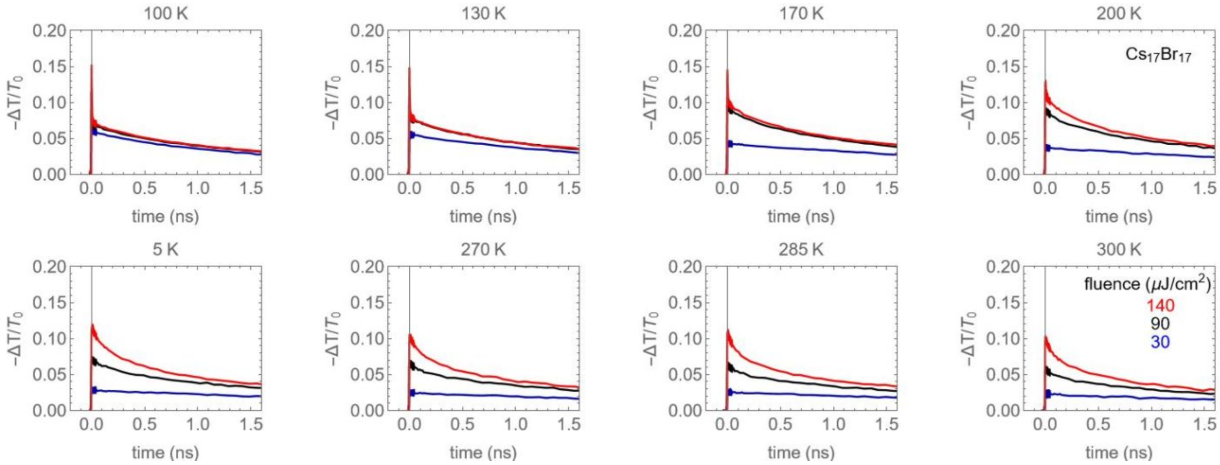

Figure S16. Raw transient THz conductivity data for the $Cs_{17}Br_{17}$ sample.

7. References

(1) Saliba, M.; Correa-Baena, J.-P.; Wolff, C. M.; Stolterfoht, M.; Phung, N.; Albrecht, S.; Neher, D.; Abate, A. How to Make over 20% Efficient Perovskite Solar Cells in Regular (*n–i–p*) and Inverted (*p–i–n*) Architectures. *Chem. Mater.* **2018**, *30* (13), 4193–4201. https://doi.org/10.1021/acs.chemmater.8b00136.
